# Chemical mixtures and birth weight: comparison of multipollutant models in the Maternal-Infant Research on Environmental Chemicals (MIREC) study

**DOI:** 10.1186/s12940-026-01300-z

**Published:** 2026-05-22

**Authors:** Susan MacPherson, Jillian Ashley-Martin, Joseph M. Braun, Youssef Oulhote, Tye E. Arbuckle

**Affiliations:** 1 Population Studies Division, Environmental Health Science and Research Bureau, Health Canada, Ottawa, ON Canada; 2 Department of Epidemiology, Brown University, Providence, RI USA; 3 Department of Environmental Medicine and Public Health, Icahn School of Medicine at Mount Sinai, New York, NY USA

## Abstract

Fetal growth is a critical health outcome influenced by prenatal exposure to environmental chemicals, particularly endocrine-disrupting chemicals (EDCs). Studies have shown that pregnant women are simultaneously exposed to multiple chemicals, illustrating the need for methods that can examine the health effects of cumulative exposures. We compared four statistical methods—Principal Component Analysis (PCA), k-means clustering, Weighted Quantile Sum Regression (WQSR), and Bayesian Kernel Machine Regression (BKMR), —to identify associations between the mixture of chemicals and birth weight z-scores, including phthalate metabolites, bisphenol A, bisphenol A alternatives, triclosan, organophosphate pesticides, arsenic species, glyphosate and its breakdown product aminomethylphosphonic acid (AMPA), solvent metabolites, organophosphate flame retardants, fluoride, polybrominated diphenyl ethers, polychlorinated biphenyls (PCBs), organochlorine pesticides (OCs), cotinine, per- and polyfluoroalkyl substances (PFAS), and five metals.

Our complete case analysis investigated the potential effects of a mixture of 46 chemicals on birth weight z-score, using 1127 mother-infant pairs from the Maternal-Infant Research on Environmental Chemicals (MIREC) study.

PCA showed a significant inverse association between birth weight z-score and a component with loadings highest for PCBs (-0.035, 95%CI: (-0.068, -0.002)) using multivariable linear regression. The k-means analysis revealed distinct clusters of chemical profiles associated with lower birth weight z-score (-0.17, 95% CI: (-0.34, -0.01)) using multivariable linear regression, and primarily composed of arsenic (As), mercury (Hg), lead(Pb), ΣOC Chlordane and the PCBs, WQSR showed an inverse association with the overall mixture index (-0.065; CI: (-0.171, 0.04) driven mostly by Aroclor 1260, ΣOC Chlordane, glyphosate, and PCB180. BKMR highlighted that the birth weight z-score was 0.054 (-0.100, 0.209)when all chemicals in the 25^th^percentile were compared to their medians, which decreased to -0.04 (-0.219, 0.14)when all chemicals in the 75th percentile were compared to their median values. After stratification by infant sex, associations tended to be larger in magnitude in females.

We observed, according to all four approaches, that ΣOC Chlordane, ΣOC Insecticides, Aroclor 1260, dimethylarsinic acid, Pb, PCB170 and PCB180 were most often associated with decreased birth weight. These findings underscore the potential adverse effects of chemical mixtures on birth weight, the usefulness of using multiple methods, and suggest the need for continued research for evaluating cumulative environmental exposures in prenatal health outcomes.

**Supplementary Information:**

The online version contains supplementary material available at 10.1186/s12940-026-01300-z.

## Introduction

Birth weight is among the most studied health outcomes in environmental epidemiology. Extreme low or high birth weight is a well-known risk factor for neonatal mortality and various morbidities in infancy, adolescence, and adulthood [35]. Evidence suggests that prenatal exposures to environmental chemicals, and in particular, endocrine disrupting chemicals (EDCs) during critical windows of development may have lasting effects throughout life [20]. The group of molecules identified as endocrine disruptors include synthetic chemicals used as industrial solvents or lubricants and their by-products, plastic compounds, plasticizers, pesticides, pharmaceutical agents, household products, and personal care and cosmetic products [19]. Metals such as cadmium, lead, mercury, and arsenicas well as environmental tobacco smoke, may also have endocrine disrupting properties [38, 51]. There is an understanding that chemicals can act together via common mechanisms and that pregnant women are exposed to multiple chemicals simultaneously [9, 25, 37, 60]. For example, EDCs such as dioxins, PCBs, polybrominated biphenyls (PBBs) and pesticides such as p,p'-dichlorodiphenyltrichloroethane (DDT), are thought to mimic natural steroid hormones and interact with hormone receptors. Heavy metals may have estrogenic activity, while several other classes of EDCs act as androgens or antiandrogens and as thyroid hormone receptor agonists or antagonists [20]. For these reasons, there is a move towards multipollutant models in environmental epidemiology and depending on the context and the goal of the research there are multiple valid methods that are available.

Broadly speaking, when examining chemical mixtures, we are interested in assessing the overall cumulative exposure, the effects of the individual compounds, and whether interactions are present [13]. The methods used to answer these questions can be classified into three groups: dimension reduction, variable selection, and grouping of observations. Some methods are preformed in an unsupervised way, where only the associations between exposures are considered, or in a supervised way, which additionally takes into account the association with the outcome [53]. There is no single method that has been established as the best method to quantify the impact of chemical mixtures on human health and studies could benefit from comparing results from multiple methods [13]. Most previous studies only considered one or two statistical mixture methods, so we aimed to compare four distinct techniques (i.e., PCA, k-means, WQSR and BKMR) that covered each type of method and utilized either unsupervised or supervised approaches. PCA is an unsupervised, dimension reduction method that transforms correlated exposures into a smaller set of independent factors that can then be analysed with birth weight [39]. One previous study used PCA to investigate the effects of prenatal exposure to a mixture of heavy metals, bisphenol, phthalates and air pollutants, on birth weight. They found that factors relating to ambient particulate matter and nitrogen dioxide exposure in early pregnancy and Pb and Hg in late pregnancy were associated with reduced birth weight [41]. Another study included metals, PCBs, OCs and PFAS in their analysis of chemical mixtures and birth weight and found that the exposure factor loaded by cadmium and arsenic was associated with reduced birth weight [25]. K-means is another unsupervised method where observations with similar exposure profiles are grouped together and the cluster assignments can be analysed in relation to birth weight using linear regression [53]. A prior study investigating prenatal exposure to a mixture of 43 chemicals and birth weight, compared PCA with k-means clustering and found that the results agreed, but argued that future studies should use both supervised and unsupervised methods, to ensure that their results are not dependant on the chosen mixtures method [34].

WQSR is a supervised method that quantizes the exposures to construct a singular index, taking into account the exposure values and weights. The derived index is used to identify the chemicals of concern and estimate the association with birth weight [55]. A previous study using WQSR found the estimate of a mixture of 16 metals was negatively related to birthweight z-score overall, and the major contributors to the mixture index were copper, nickel, manganese and cadmium [60]. A similar method called quantile g-computation (Qgcomp), shares the simplicity of inference with WQSR but does not allow for identifying individual components that contribute most to the mixture effect. Qgcomp was previously used to evaluate the overall mixture effect of 11 phthalate metabolites, 3 bisphenols, and 5 dialkylphosphate (DAP) metabolites, found that infants in the fourth quartile of exposure had lower birth weight in comparison with those in first quartile [56].

Also considered a supervised approach, Bayesian kernel machine regression (BKMR) is a variable selection method that can identify important chemicals and evaluate the joint effect of chemicals on birth weight, while identifying nonlinear and non-additive relationships within and between chemical interactions [9, 10]. Previously, BKMR has been used to evaluate associations between birth weight and a phthalate mixture and they identified that monoethyl phthalate and mono(2-ethylhexyl) phthalate concentrations were linearly related to lower birth weight. These results were corroborated by also performing PCA, which identified two principal components inversely associated with birth weight [16]. In a pooled BKMR analysis of metal mixtures, another study found that antimony, mercury, and tin were inversely and linearly associated with BW for GA, while a positive linear association was identified for nickel [30]. Another study, examining seven classes of chemical mixtures similar to those identified in our analysis, found that exposure to PCB and PFAS both displayed inverse associations with birth weight [61]. Other Bayesian methods have also been a popular choice among researchers, including Bayesian factor analysis (BFA), Bayesian hierarchical linear models (BHLM) and Bayesian structured additive regression (BSAR) [6, 40, 59, 61].

There is no one widely accepted statistical approach recommended in mixture studies [27]. Instead, similar to our analysis, several previous studies have used different statistical approaches on the same dataset [22, 26, 42]. As is the case with much of the current epidemiological investigation of chemical mixtures, we used an exploratory statistical approach to discover profiles of chemicals that are predictive of birth weight. The objective of this analysis was to compare four unique statistical approaches, PCA, k-means, WQSR and BKMR, by examining the potential effects of in utero exposure to a mixture of 46 chemicals on birth weight in singleton live born infants from a pan-Canadian pregnancy cohort. The ultimate goal of our objective is to try to answer the research question while comparing the application of different methods to evaluate health effects of chemical mixtures.

## Methods

### Study participants

The Maternal-Infant Research on Environmental Chemicals (MIREC) study is a Canadian cohort of 2,001 pregnant women, recruited from 10 Canadian cities between 2008 and 2011 [1]. Trained research staff administered questionnaires to participants about their demographic information, medical history, and lifestyle,medical information about the pregnancy was abstracted from medical charts. Out of the 2001 women who consented to the study, 1859 women gave birth to a live singleton infant. Of those, 1217 had measurements for the complete set of chemicals, from urine and blood collection during the first trimester, and had consented to having their specimens stored in a biobank for future research. From this, we removed 6 with missing values for infant weight, 1 for maternal education, and 83 for maternal BMI, resulting in 1127 mother-infant pairs for our complete case analysis. The MIREC Study received ethics approval from the Health Canada Research Ethics Board and ethics committees at all recruitment sites. All participants provided informed consent.

### Chemical biomarkers

An extensive list of proven and suspected EDCs was measured in maternal urine and blood samples during the 1 st trimester. Analytical methods have been described in detail elsewhere [2–5, 7, 8, 12, 23, 24, 52]. Of the 143 individual chemicals we considered, 66 were excluded for being detected in less than 50% of the samples. Of the 77 remaining, chemicals within a given group that share a common source or metabolic pathway and were highly correlated were summed, resulting in 46 chemical compounds, forming 15 groups (Table 1).

**Table 1:** Chemical groups and analytes, MIREC Study

Chemical Group	**Abbreviation**	**Chemical**
Arsenic species		
	DMAA	dimethylarsinic acid
Bisphenol A Alternatives/Analogues		
	BP44	bisphenol 4,4’
	BPE	bisphenol E
	BPF	bisphenol F
	BPS	bisphenol S
	DHDPE	4,4'-dihydroxydiphenyl ether
Fluoride		
	FLD	fluoride
Herbicides		
	AMPA	aminomethylphosphonic acid
	GLYP	glyphosate
Metals		
	As	arsenic
	Cd	cadmium
	Hg	mercury
	Mn	manganese
	Pb	lead
Organochlorine Compounds		
	ΣOC_Chlordane	Σ OXYCHLOR, TRANSNONA, CISNONA
	OXYCHLOR	oxychlordane
	TRANSNONA	trans-nonachlor
	CISNONA	cis-nonachlor
	ΣOC_Insecticides	Σ HEXACHLOR, BBHC, DDE, DDT
	HEXACHLOR	hexachlorobenzene
	BBHC	beta -hexachlorocylcohexane
	DDE	p,p'-dichlorodiphenyldichloroethylene
	DDT	p,p'-dichlorodiphenyltrichloroethane
Organophosphate Flame Retardants		
	BDCliPrP	bis(1,3-dichloro-2-propyl) phosphate
	DBP	di-n-butyl phosphate
	tb_DPhP	tert-butyl-phenyl phenyl phosphate
	ΣTCiPP	
	BCliPrP	bis-(2-chloroisopropyl) phosphate
	OHiPrBCliPrP	1-hydroxyl-2-propyl bis(1-chloro-2-propyl) phosphate
	ΣTCrP	
	BoTyP	bis o-cresyl phosphate
	BpTyP	bis p-cresyl phosphate
	BmTyP	bis m-cresyl phosphate
	ΣTECP	
	BClEtOHEtP	bis(2-chloroethyl) 2-hydroxyethyl phosphate
	BClEtP	bis-(2-chloroethyl) hydrogen phosphate
	ΣTPhP	
	DPhP	diphenyl phosphate
	mOH_TPhP	meta isomers of OH-TPhP
	p_iPr_DPhP	isopropyl diphenyl phosphate
Organophosphate Pesticides	ΣDEAP	Σ DEP, DETP, DEDTP
	DEDTP	diethyldithiophosphate
	DEP	diethylphosphate
	DETP	diethylthiophosphate
	ΣDMAP	Σ DMP, DMTP, DMDTP
	DMDTP	dimethyldithiophosphate
	DMP	dimethylphosphate
	DMTP	dimethylthiophosphate
PolyBrominated Diphenyl Ethers		
	ΣPBDE	Σ PBB153,BDE100, PBDE153, PBDE28, PBDE33, PBDE47, PBDE99
	PBB153	2,2',4,4',5,5'-hexabromobiphenyl
	PBDE100	2,2',4,4',6-pentabromodiphenyl ether
	PBDE153	2,2',4,4',5,5' -hexabromodiphenyl ether
	PBDE28	2,4,4'-tribromodiphenyl ether
	PBDE33	2,3',4'-tribromodiphenyl ether
	PBDE47	2,2',4,4'—tetrabromobiphenyl
	PBDE99	2,2',4,4',5-pentabromodiphenyl ether
PolyChlorinated Biphenyls		
	PCB170	2,2',3,3',4,4',5-heptachlorobiphenyl
	PCB180	2,2',3,4,4',5,5'-heptachlorobiphenyl
	ΣPCB	ΣPCB105, PCB118, PCB156, PCB167
	PCB105	2,3,3',4,4'-pentachlorobipheny
	PCB118	2,3',4,4',5-pentachlorobiphenyl
	PCB156	2,3,3',4,4',5-hexachlorobiphenyl
	PCB167	2,3',4,4',5,5'-hexachlorobiphenyl
	Aroclor 1260	Aroclor 1260: (PCB153 + PCB138) × 5.2
	PCB138	2,2',3,4,4',5'-hexachlorobiphenyl
	PCB153	2,2',4,4',5,5'-hexachlorobiphenyl
Per- and polyfluoroalkyl Substances		
	PFHxS	perfluorohexane sulfonate
	PFOA	perfluorooctanoic acid
	PFOS	perfluorooctane sulfonate
Phenols		
	TCS	Total triclosan
	BPA	Total bisphenol A
Phthalates		
	MBzP	mono benzyl phthalate
	MCPP	mono-3-carboxypropyl phthalate
	MEP	mono ethyl phthalate
	MMP	mono-methyl phthalate
	ΣDEHP	Σ MEHHP, MEPH, MEOHP, MCMHP, MECPP
	MEHHP	mono-(2-ethyl-5-hydroxyhexyl) phthalate
	MEHP	mono-2-ethylhexyl phthalate
	MEOHP	mono-(2-ethyl-5-oxohexyl) phthalate
	MCMHP	mono(2-carboxy-methylhexyl) phthalate
	MECPP	mono(2-ethyl-5-carboxy-pentyl) phthalate
	ΣDiBP	Σ 2OHMIBP, MiBP
	2OHMIBP	2-hydroxy-mono-iso-butyl phthalate
	MiBP	mono-iso-butyl phthalate
	ΣDiDP	Σ MCiNP, MHiDP, MiDP, MOiDP
	MCiNP	mono-(2,7-methyl-7-carboxyheptyl) phthalate
	MHiDP	mono-(2-propyl-6-hydroxy-heptyl) phthalate
	MiDP	mono-iso-decyl phthalate
	MOiDP	mono-(2-propyl-6oxoheptyl) phthalate
	ΣDiNP	Σ MCiOP, MHiNP, MiNP, MOiNP
	MCiOP	mono(carboxy-isooctyl) phthalate
	MHiNP	mono(hydroxy-isononyl) phthalate
	MiNP	mono-isononyl phthalate
	MOiNP	mono(oxo-isononyl) phthalate
	ΣDnBP	Σ MHBP, MnBP
	MHBP	mono-3-hydroxy-n-butyl phthalate
	MnBP	mono-n-butyl phthalate
Solvents		
	ΣNEP	Σ 5HNMP, 2HMSI
	5HNMP	5-hydroxy-n-methyl-2-pyrrolidone
	2HMSI	2-hydroxy-n-methylsuccinimide
Tobacco Metabolite		
	Cotinine	cotinine

All urinary chemical biomarker concentrations should be adjusted for urinary dilution, typically using urinary creatinine or the specific gravity (SG) of the urine sample. Due to the physiological changes during pregnancy, SG adjustment may be a favorable approach for correcting urinary dilution [43]. The SG adjusted concentrations were calculated using the following formula: Pc = Pi [(SGm– 1)/(SGi – 1)], where Pc is the SG-standardized metabolite concentration (μg/L), Pi is the observed metabolite concentration, SGi is the specific gravity of the urine sample and SGm is the median SG for the maternal cohort (adapted from Just et al., [32] and Duty et al., [21]). To account for the variability in plasma lipid levels, PBDEs, PCBs and OCs were standardized by dividing the original concentration by total plasma lipid concentrations and expressed in units of mg/kg lipids. The molar sums were calculated by dividing each metabolite concentration by its molecular weight and then summing them together.

### Assessment of fetal growth

Infant sex, birth weight in grams and gestational age were recorded at delivery from a chart review. Several previous studies have used birth weight, along with gestational age as a measure of fetal growth [25, 40, 41, 59]. However, gestational age may be on the causal pathway between chemical exposure and birth weight [57], and recent studies have taken this into account and used a gestational age adjusted birth weight as the measure of fetal growth [6, 16, 29–31, 34, 60], Van den [56, 61]. Our outcome of interest was birth weight-for-gestational age z-scores, calculated based on the sex and gestational age specific standards from Canadian birth data from 2008–2011 [44].

### Covariates

Potential confounders were selected using a directed acyclic graph (DAG) [50, 58] based on previous studies of chemical exposure, covariates, and fetal growth (Figure S1) [6, 16, 17, 31, 59]; (Larzarevic et al., 2022). The following maternal covariates were determined: education, parity, country of birth, age, and pre-pregnancy body mass index (BMI).

### Statistical analysis

Considering only chemicals that had greater than a 50% detection rate, machine readings provided by the laboratory were used for the concentrations below the level of detection (LOD); unless they were not available, then results were replaced by LOD/√2, as has been done previously [56]. All chemicals were log_2_-transformed to reduce the influence of outliers. Pearson’s correlation coefficients were used to examine the correlations among the log_2_-transformed chemicals. To put the chemicals on a common scale, prior to performing the analyses, z-scores for each chemical were calculated, by subtracting the mean and dividing by the standard deviation.

Descriptive statistics for the maternal demographic factors and the standardized chemicals were calculated. Multivariable linear regressions were used to model the associations between birth weight z-score and each of the chemicals, separately. The final models included the following covariates: maternal education, parity, country of birth, age, and pre-pregnancy BMI.

Following that, four mixture methods were deployed, and all models included the same covariates.

#### Principal Component Analysis (PCA)

PCA is a common unsupervised method for dimension reduction. It reduces multidimensional exposure data to several orthogonal, uncorrelated principal components (PCs). The variance–covariance matrix of the original data is decomposed into a diagonal matrix formed from eigenvalues, which represent the variance explained by each PC. Another matrix is formed by eigenvectors, whose elements are the weights that describe how much each variable contributes to a particular PC. Each PC is calculated as a linear combination of the original predictors, resulting from the weights that maximize the overall explained variability. A PC represents a mixture of chemicals, each contributing with different loadings [53]. The sign of a loading indicates whether a variable and a PC is positively or negatively correlated [28]. What constitutes a large loading is found by calculating$\sqrt{1/p}$, where $p$ is the total number of chemicals, which in our case is 0.147.

The number of PCs were chosen using a point where the proportion of explained variance from additional factors would add relatively little to the information. PC scores were calculated by multiplying the original chemical concentration z-scores by that chemical’s PCA loading [33]. The scores of the chosen PCs were treated as continuous variables and used in a multiple linear regression model, in place of the original exposure variables, while adjusting for covariates. The effect estimates can be interpreted as the change in birthweight z-score for every standard deviation increase in the PC, while adjusting for covariates.

#### K-means clustering

K-means is the most widely used clustering method, and it falls into the partitioning-based category [53]. It is a method in which observations are sequentially grouped according to a proximity measure, known as the Euclidean distance.

The k-means clustering algorithm randomly assigns k initial centres then iteratively assigns each observation to the nearest centre. This process repeats until a new iteration no longer re-assigns any observations to a new cluster and each participant is classified into a final cluster based on their distinctive exposure profiles. The number of clusters is assigned a priori based on subject matter knowledge. Because there was no prior knowledge, the elbow method shows a point of inflection where the sum of squared distance between data points and the centres is minimal. A categorical variable identifying the clusters was used in a multiple linear regression model, in place of the original exposure variables, while adjusting for covariates.

#### Weighted Quantile Sum Regression (WQSR)

WQSR is a popular supervised dimension reduction method that was created to provide both estimates of mixture health effects and indicators of important mixture components. Technical details on the WQSR method can be found elsewhere [14]. Briefly, the association between chemical quantiles and the outcome produces weights that range from 0 to 1 and sum to 1, describing the relative contributions of each chemical to the overall association. A WQS index is used to estimate an overall mixture effect of all 46 chemical compounds. WQSR assumes an either positive or negative linear association between the exposure index and outcome of interest. We employed a two-index model which allows for both positive and negative directions and provides separate representation of the relative importance of elements for overall positive or negative directional effects [47]. The method assumes that there are no interactions among the exposures incorporated into the WQSR index [14]. The index is used as a variable in a multiple linear regression model, along with the covariates, to estimate the association between the combined chemicals and the outcomes. Effect estimates generated from WQS models represent the change in birth weight z-score for a particular percentile increase in the environmental exposure index [60]. Similar to the PCA, a cut-off criterion for what constitutes a large weight was set to a weight of 2.17%, a value consistent with equal weighting (1/46 chemicals) [55].

Furthermore, we used a repeated holdout validation technique to address the problems associated with partitioned data, or training and testing on the same dataset. Specifically, repeated holdout validation combines cross validation and bootstrap resampling [55], where the data were randomly partitioned, with replacement, 100 times and WQS regression was repeated on each set. With 100 bootstraps per repetition, weights were estimated 10,000 times [14, 55].

#### Bayesian Kernel Machine Regression (BKMR)

BKMR is a non-parametric method that can be used to evaluate the joint effect of chemicals on birth weight z-score. To evaluate the overall mixture effect, BKMR estimates a high-dimensional set of predictors, and the outcome is modeled using a flexible exposure–response function estimated with a gaussian kernel. The default noninformative priors were used for analyses [10]. BKMR implements Markov Chain Monte Carlo (MCMC), an iterative algorithm for the estimation of the exposure–response function. For our analysis we used 50,000 iterations, where the first 5000 iterations were discarded as a burn in period, and every 50th iteration was kept, to reduce autocorrelation in the sample. We examined the convergence of the algorithm using trace plots for selected parameters, which exhibited the stable fluctuations around a central value, indicating model convergence. The resulting function is used to represent the risk associated with all exposures at a particular quantile vs. the median and allows for exposure-outcome associations that are non-linear, and for the analysis of the interactions between exposures.

Since some of the chemicals in our analysis were highly correlated or shared a common source or metabolic pathway, we identified groupings for each compound, as previously specified. The hierarchical variable selection approach addresses the issue of multicollinearity by placing highly correlated pollutants into the same group [10, 16, 31, 61]. BKMR estimates the posterior inclusion probability (PIP) for each chemical group, as well as the conditional PIPs for the chemicals within each group. PIPs range from 0 to 1 with the higher PIPs indicating greater variable importance [9, 31].

Lastly, because previous studies have reported sex-specific associations between birth outcomes and a number of environmental chemicals [6, 25, 30, 31, 34, 59], we conducted additional sex-specific analysis for males and females separately. A sensitivity analysis that excluded preterm newborns (< 37 gestational weeks) was also performed. All statistical analyses were conducted using SAS 9.4 (SAS Institute, Cary, North Carolina, USA) and R version 3.3.2 [45]. The linear modelling, PCA and k-means analysis used the functions lm(), prcomp() and kmeans() from the stats package [45]. WQSR was performed using the gwqs() function from the gWQS package, [46] and BKMR used the kmbayes() function from the bkmr package [11].

## Results

Demographic characteristics for the 1127 mother-infant pairs in our complete case analysis, as well as the 733 participants who were not included, are provided in Table 2. The women were mostly born in Canada (80%), identified as white (84%), with at least an undergraduate degree (64%), were multiparous (70%), had a mean pre-pregnancy BMI of 25 kg/m^2^ and averaged 32 years old. The mean infant birth weight was 3454 g for the overall sample, 3511 g for male infants and 3392 g for female infants. The mean gestational age was 39.4 weeks, with approximately 6% of infants born before 37 weeks’ gestation.

**Table 2:** Characteristics of participants in the MIREC study

Characteristic	Participants included in the mixture analysis (*n = *1127)		Participants not Included in the mixture analysis (*n = *733)	
	**No. of participants**	**Percentage (%)**	**No. of participants**	**Percentage (%)**
Maternal Education				
≤ High School	88	7.81	73	9.99
Some College	60	5.32	38	5.20
College/Trade School Diploma	249	22.09	190	25.99
University Degree	730	64.77	430	58.82
Missing			2	
Household Income ($)				
< = 50,000	159	14.75	159	22.84
50,001–100,000	455	42.21	285	40.95
≥ 100,000	464	43.04	252	36.21
Missing	49		37	
Mother’s Country of Birth				
Canada	902	80.04	603	82.26
Other	225	19.96	130	17.74
Mother’s Race				
White	949	84.21	606	82.67
Other	178	15.79	127	17.33
Parity				
Nulliparous	335	29.72	200	27.29
Multiparous	792	70.28	533	72.71
	Range	µ (s.d.)	Range	µ (s.d.)
Maternal Age (years)	[17, 48]	32.36 (5.01)	[18, 47]	32.83 (5.2)
Pre-pregnancy Body Mass Index (kg/m2)	[15.8, 58.6]	24.82 (5.37)	[15.63, 67.0]	25.13 (5.83)
Missing			138	

Descriptive statistics of all SG or lipid standardized chemicals measured in first trimester maternal urine or plasma/whole blood samples are shown in Table S1. For comparison, the descriptive statistics for the 733 participants not included in our sample are shown in Table S2. From the heat map of Pearson’s correlations, we observed mostly positive correlations between log-transformed chemicals, with the strongest correlations ranging from 0.69 to 0.94 among the PCBs (Figure S2).

In multivariable linear regression models of individual chemicals, we observed statistically significant decreases in birth weight for a twofold increase in ∑OC chlordane and Pb. However, when the analysis was stratified by sex, the only significant results were found in female infants, with a decrease in birth weight z-score (95% CI) of −0.087 (−0.17, −0.004) for BPE, −0.108 (−0.194, −0.022) for DHDPE, −0.1 (−0.195, −0.005) for ∑OC chlordane, and −0.087 (−0.171, −0.002) for BDCLIPRP (Table 3). When we repeated the analysis to only include full term infants, the results were largely comparable (data not shown).

**Table 3:** Associations between birth weight z-score and chemicals using separate linear regression models, MIREC Study

Chemical	**Overall (*n = *1127)**	**Males (*n = *589)**	**Females (*n = *538)**
	**Estimate**^**a**^** (95% CI)**	**Estimate (95% CI)**	**Estimate (95% CI)**
DMAA	−0.054 (−0.113, 0.005)	−0.019 (−0.1, 0.062)	−0.082 (−0.168, 0.005)
BP44	−0.053 (−0.113, 0.007)	−0.023 (−0.104, 0.059)	−0.071 (−0.159, 0.017)
BPE	−0.037 (−0.094, 0.021)	−0.014 (−0.095, 0.067)	**−0.087 (−0.17, −0.004)**
BPF	−0.013 (−0.071, 0.046)	0.026 (−0.055, 0.107)	−0.005 (−0.09, 0.08)
BPS	−0.027 (−0.085, 0.03)	0.025 (−0.056, 0.106)	0.006 (−0.078, 0.089)
DHDPE	−0.034 (−0.094, 0.027)	0.007 (−0.074, 0.088)	**−0.108 (−0.194, −0.022)**
AMPA	0.029 (−0.029, 0.087)	−0.021 (−0.102, 0.06)	0.032 (−0.051, 0.116)
GLYP	−0.019 (−0.077, 0.038)	0 (−0.082, 0.082)	0.028 (−0.055, 0.111)
As	−0.042 (−0.1, 0.016)	0.031 (−0.05, 0.112)	−0.039 (−0.124, 0.045)
Cd	−0.049 (−0.108, 0.011)	−0.015 (−0.096, 0.066)	−0.051 (−0.137, 0.035)
Hg	0.004 (−0.059, 0.067)	0.063 (−0.019, 0.144)	−0.033 (−0.125, 0.06)
Mn	0.008 (−0.05, 0.065)	0 (−0.081, 0.082)	0.008 (−0.076, 0.092)
Pb	**−0.062 (−0.122, −0.001)**	−0.056 (−0.138, 0.026)	−0.075 (−0.163, 0.013)
ΣOC Chlordane	**−0.078 (−0.142, −0.014)**	0.003 (−0.078, 0.084)	-**0.1 (−0.195, −0.005)**
ΣOC Insecticides	−0.048 (−0.119, 0.022)	−0.024 (−0.105, 0.057)	−0.057 (−0.162, 0.048)
BDCliPrP	−0.033 (−0.092, 0.026)	0.057 (−0.024, 0.138)	**−0.087 (−0.171, −0.002)**
DBP	−0.004 (−0.062, 0.053)	0.005 (−0.076, 0.086)	0.011 (−0.072, 0.094)
tb_DPhP	0.03 (−0.028, 0.087)	0.048 (−0.033, 0.129)	0.071 (−0.012, 0.154)
ΣTCiPP	−0.037 (−0.095, 0.021)	0 (−0.082, 0.081)	−0.013 (−0.097, 0.07)
ΣTCrP	0.026 (−0.032, 0.083)	0.101 (0.02, 0.181)	0.057 (−0.026, 0.14)
ΣTECP	−0.016 (−0.076, 0.043)	−0.044 (−0.125, 0.037)	0.004 (−0.082, 0.089)
ΣTPhP	0.051 (−0.006, 0.109)	−0.023 (−0.104, 0.058)	0.017 (−0.067, 0.101)
ΣDEAP	−0.022 (−0.08, 0.035)	0.05 (−0.03, 0.131)	−0.028 (−0.111, 0.056)
ΣDMAP	−0.01 (−0.068, 0.047)	−0.039 (−0.12, 0.042)	0.006 (−0.078, 0.09)
ΣPBDE	−0.013 (−0.071, 0.045)	0.05 (−0.03, 0.131)	0.018 (−0.066, 0.102)
Aroclor 1260	−0.056 (−0.129, 0.016)	0.011 (−0.07, 0.092)	−0.069 (−0.178, 0.04)
PCB170	−0.043 (−0.114, 0.028)	−0.018 (−0.1, 0.063)	−0.087 (−0.191, 0.017)
PCB180	−0.05 (−0.126, 0.027)	−0.043 (−0.124, 0.038)	−0.07 (−0.185, 0.044)
ΣPCB	−0.011 (−0.074, 0.052)	−0.028 (−0.11, 0.053)	−0.049 (−0.142, 0.045)
PFHxS	0.008 (−0.051, 0.068)	−0.059 (−0.14, 0.022)	−0.007 (−0.092, 0.079)
PFOA	−0.011 (−0.072, 0.05)	0.043 (−0.039, 0.124)	−0.044 (−0.133, 0.045)
PFOS	0.013 (−0.046, 0.071)	−0.016 (−0.097, 0.065)	0.005 (−0.079, 0.089)
BPA	−0.019 (−0.077, 0.039)	0.053 (−0.028, 0.134)	−0.035 (−0.118, 0.049)
TCS	0.031 (−0.026, 0.089)	0.1 (0.019, 0.181)	0.017 (−0.066, 0.101)
MBzP	0.05 (−0.009, 0.109)	0.004 (−0.077, 0.084)	0 (−0.086, 0.087)
MCPP	−0.029 (−0.086, 0.029)	0.025 (−0.056, 0.106)	−0.046 (−0.129, 0.037)
MEP	0.028 (−0.029, 0.086)	−0.068 (−0.149, 0.013)	0.019 (−0.066, 0.103)
MMP	−0.032 (−0.09, 0.026)	−0.026 (−0.107, 0.055)	−0.057 (−0.14, 0.027)
ΣDEHP	−0.03 (−0.088, 0.027)	0.032 (−0.049, 0.113)	−0.01 (−0.094, 0.073)
ΣDiBP	−0.05 (−0.109, 0.009)	0.018 (−0.063, 0.099)	−0.068 (−0.155, 0.019)
ΣDiDP	−0.012 (−0.069, 0.046)	−0.014 (−0.095, 0.067)	−0.019 (−0.103, 0.064)
ΣDiNP	−0.006 (−0.063, 0.052)	−0.012 (−0.094, 0.07)	0.043 (−0.04, 0.126)
ΣDnBP	−0.01 (−0.068, 0.048)	−0.02 (−0.103, 0.062)	−0.032 (−0.116, 0.052)
ΣNEP	0.063 (0.006, 0.121)	0.046 (−0.035, 0.127)	0.056 (−0.027, 0.139)
Cotinine	−0.027 (−0.088, 0.035)	−0.005 (−0.086, 0.076)	−0.046 (−0.135, 0.044)
FLD	0.035 (−0.023, 0.092)	0.05 (−0.03, 0.131)	−0.026 (−0.11, 0.057)

^a^Regression coefficient represents a change in birth weight z-score per doubling of chemical concentration z-score

Bold indicates *p*-value <0.05

Models were adjusted for maternal education, parity, maternal country of birth, age and pre-pregnancy BMI

### PCA

**Fig. 1:**
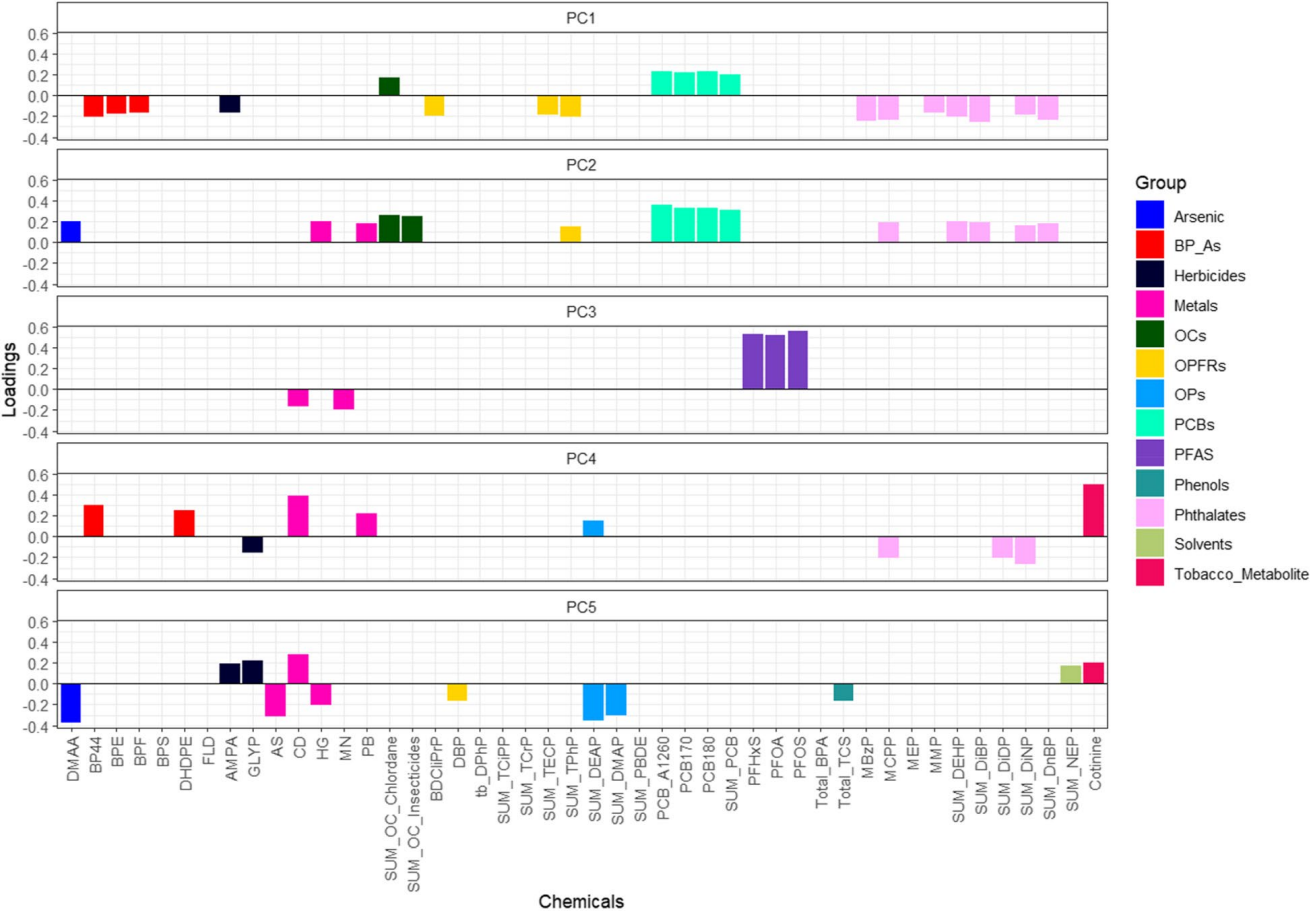
PCA loadings, MIREC Study. Models were adjusted for maternal education, parity, maternal country of birth, age and pre-pregnancy BMI

Five PCs, which accounted for 55% of the total variance, were retained. To interpret how the exposure variables contribute to each of the principal components, we looked at the loadings larger than our cut-off criterion of 0.147. Regardless of whether the analysis was on the overall sample or separated by infant sex, the PCs had very similar loadings. The loadings of each chemical for the five PCs are presented in Fig. 1.

The PC regression analysis, for the overall sample, showed a significant inverse association between birth weight z-score and PC2, −0.035, 95%CI: (−0.068, −0.002), which had strong positive loadings for DMAA, Hg, Pb, ΣOC Chlordane, ΣOC Insecticides, ΣTPHP, Aroclor 1260, PCB170, PCB180, ΣPCB, MBzP, ΣDEHP, ΣDIBP, ΣDINP, and ΣDNBP. An inverse association was found for female infants and PC2, −0.048, 95%CI: (−0.093, −0.002), which had positive loadings for DMAA, BPE, Hg, Pb, ΣOC Chlordane, ΣOC Insecticides, ΣTPHP, Aroclor 1260, PCB170, PCB180, ΣPCB, MBZP, MCPP, ΣDEHP, ΣDIBP, ΣDINP, and ΣDNBP. Birth weight z-score for female infants was also inversely associated with PC4, −0.075 95%CI: (−0.138, −0.011), which had additional positive loadings for BP44, DHDPE, As, Cd, DBP, ΣDEAP and Cotinine. There was a non-significant inverse association found for the male infants and PC2, with highest positive loadings for DMAA, Hg, Pb, ΣOC Chlordane, ΣOC Insecticides, Aroclor 1260, PCB170, PCB180, ΣPCB, ΣDEHP and ΣDIBP. No significant associations were found between any of the other principal components and birth weight z-score (Table 4)*.*

**Table 4:** Association between birth weight z-score and 5 principal components, MIREC Study

**Principal Component**^**a**^	**Overall (*N = *1127)**	**Males (*n = *589)**	**Females (*n = *538)**
	**Estimate**^**b**^** (95% CI)**	**Estimate**^**b**^** (95% CI)**	**Estimate**^**b**^** (95% CI)**
PC1	−0.007 (−0.035, 0.021)	0.007 (−0.03, 0.043)	0.015 (−0.03, 0.06)
PC2	**−0.035 (−0.068, −0.002)**	−0.023 (−0.07, 0.024)	**−0.048 (−0.093, −0.002)**
PC3	0.022 (−0.02, 0.063)	0.029 (−0.028 0.085)	0.007 (−0.053, 0.068)
PC4	−0.039 (−0.084, 0.005)	0.001 (−0.059, 0.061)	**−0.075 (−0.138, −0.011)**
PC5	0.002 (−0.044, 0.048)	0.006 (−0.057, 0.068)	0.02 (−0.044, 0.084)

Models were adjusted for maternal education, parity, maternal country of birth, age and pre-pregnancy BMI

^a^The 5 principal components accounted for 55% of the total variance. The loadings are shown in Fig. 1

^b^Estimates represent the mean change in birth weight z-score for a 1 unit change in the PC score

Bold indicates *p*-value <0.05

### K-Means

**Fig. 2:**
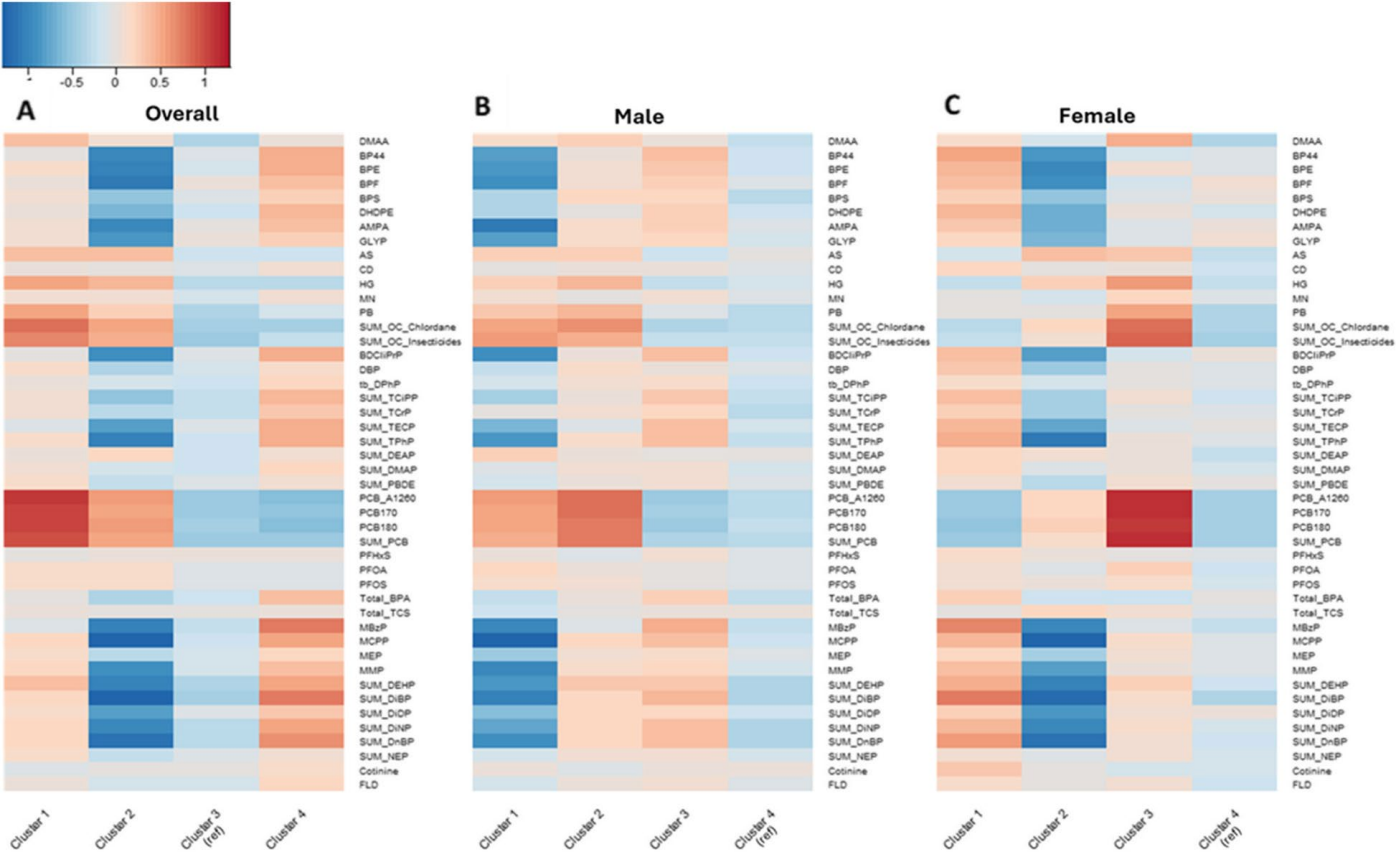
Chemical concentration z-scores by k-means cluster, MIREC Study. Models were adjusted for maternal education, parity, maternal country of birth, age and pre-pregnancy BMI. **A** Overall sample; **B** Male Infants; **C** Female infants

Based on the elbow method, 4 clusters were chosen as the optimal number of clusters. The categorical group assignments were used instead of the exposure in a linear regression model for birth weight z-score, along with all the covariates. When analysing the overall sample, using cluster 3 as the reference, cluster 1 was inversely associated with birth weight z-score, −0.171, 95% CI: (−0.337, −0.005), characterized by high levels of DMAA, As, Hg, Pb, ΣOC Chlordane, ΣOC Insecticides, Aroclor 1260, PCB170, PCB180 and ΣPCB. For female infants, when compared to reference cluster 4, cluster 1 was inversely associated with birth weight z-score with an estimate of −0.24, 95% CI: (−0.449, −0.031) and having the highest levels of BP44, DHDPE, ΣTPHP, MBZP, MCPP, ΣDIBP, ΣDNBP and Cotinine. Cluster 3 was also inversely associated with birth weight z-score −0.351, 95% CI: (−0.597, −0.105), having high concentrations of DMAA, As, Hg, Pb, ΣOC Chlordane, ΣOC Insecticides, Aroclor 1260, PCB170, PCB180, and ΣPCB. For male infants, there were non-significant associations for all clusters, when compared to the reference cluster (Table 5, Fig. 2).

**Table 5:** Differences in birth weight z-score by chemical cluster, MIREC Study

CLUSTER^a^	**Overall (*n = *1127)**	**Males (*n = *589)**	**Females (*n = *538)**
	**Estimate**^**b**^** (95% CI)**	**Estimate (95% CI)**	**Estimate (95% CI)**
Cluster 1	**−0.171 (−0.337, −0.005)**	−0.067 (−0.385, 0.252)	**−0.24 (−0.449, −0.031)**
Cluster 2	−0.167 (−0.372, 0.037)	−0.143 (−0.377, 0.09)	−0.178 (−0.465, 0.108)
Cluster 3	ref^c^	0.029 (−0.175, 0.232)	**−0.351 (−0.597, −0.105)**
Cluster 4	−0.083 (−0.23, 0.063)	ref	ref

Models were adjusted for maternal education, parity, maternal country of birth, age and pre-pregnancy BMI

^a^The chemical concentrations by cluster are shown in Fig. 2

^b^Estimates represent the mean change in birth weight z-score for each cluster when compared to reference cluster

^c^Reference cluster has the lowest chemical concentrations and the highest birth weight z-score

Bold indicates *p*-value <0.05

### WQSR

The negative WQS indices, while statistically insignificant, were inversely related to birth weight z-score for all infants (−0.065, 95% CI: (−0.171, 0.04)), male infants (−0.111 (−0.296, 0.073)), and female infants (−0.042 (−0.211, 0.127)) (Table 6). In the negative direction the weights for the overall sample, in order from largest to smallest, were above the threshold for Aroclor 1260, ΣOC Chlordane, GLYP, PCB180, MCPP, BP44, ΣDiBP, As, Pb, BDCliPrP, ΣOC Insecticides, ΣTCiPP, Cd, DMAA, Cotinine, MMP, BPE, PCB170, PFOA, ΣDiNP, DHDP. When we restricted the sample to include only males, Aroclor 1260, GLYP, PCB180, ΣDiNP, As, ΣOC Chlordane, BP44, ΣTCiPP, ΣDiBP, ΣPBDE, Pb, ΣDEHP, ΣOC Insecticides, MCPP, Cd, BPF, ΣDEAP, and DMAA, contributed to the majority of mixture association. For the females only model, high weights were observed for ΣOC Chlordane, BDCliPrP, BPE, PCB180, DHDPE, ΣDiBP, PCB170, MCPP, ΣOC Insecticides, Aroclor 1260, As, Pb, PFOA, Cd, Cotinine, DMAA, ΣDnBP, and ΣDiDP (Figure S3).

**Table 6:** Association between birth weight z-score and the WQS index, MIREC Study

	Overall (*n* = 1127)	Males (*n* = 589)	Females (*n* = 538)
	Estimate^c^ (95% CI)	Estimate (95% CI)	Estimate (95% CI)
Negative WQS index^a^	−0.065 (−0.171, 0.04)	−0.111 (−0.296, 0.073)	−0.042 (−0.211, 0.127)
Positive WQS index^b^	0.013 (−0.113, 0.139)	0.082 (−0.109, 0.272)	−0.065 (−0.227, 0.098)

^a^The negative WQS weights are shown in Figure S3

^b^The positive WQS weights are shown in Figure S4

^c^Estimates represent the mean change in birth weight z-score per quantile increase in WQS index

Models were adjusted for maternal education, parity, maternal country of birth, age and pre-pregnancy BMI

The positive direction WQS indices were related to birth weight z-score with estimates (95% CI) of 0.013 (−0.113, 0.139) for all infants and 0.082 (−0.109, 0.272) for males only. The female infant index was −0.065 (−0.227, 0.098) which may indicate that the model has failed to detect any bootstrapped coefficients in the positive direction because there is little to no signal in that direction [18]. The weights for the positive direction were found to be lower than in the negative direction, but were highest for ΣPCB, PCB 180, MBzP, ΣTPhP and AMPA, shown in Figure S4.

### BKMR

**Fig. 3:**
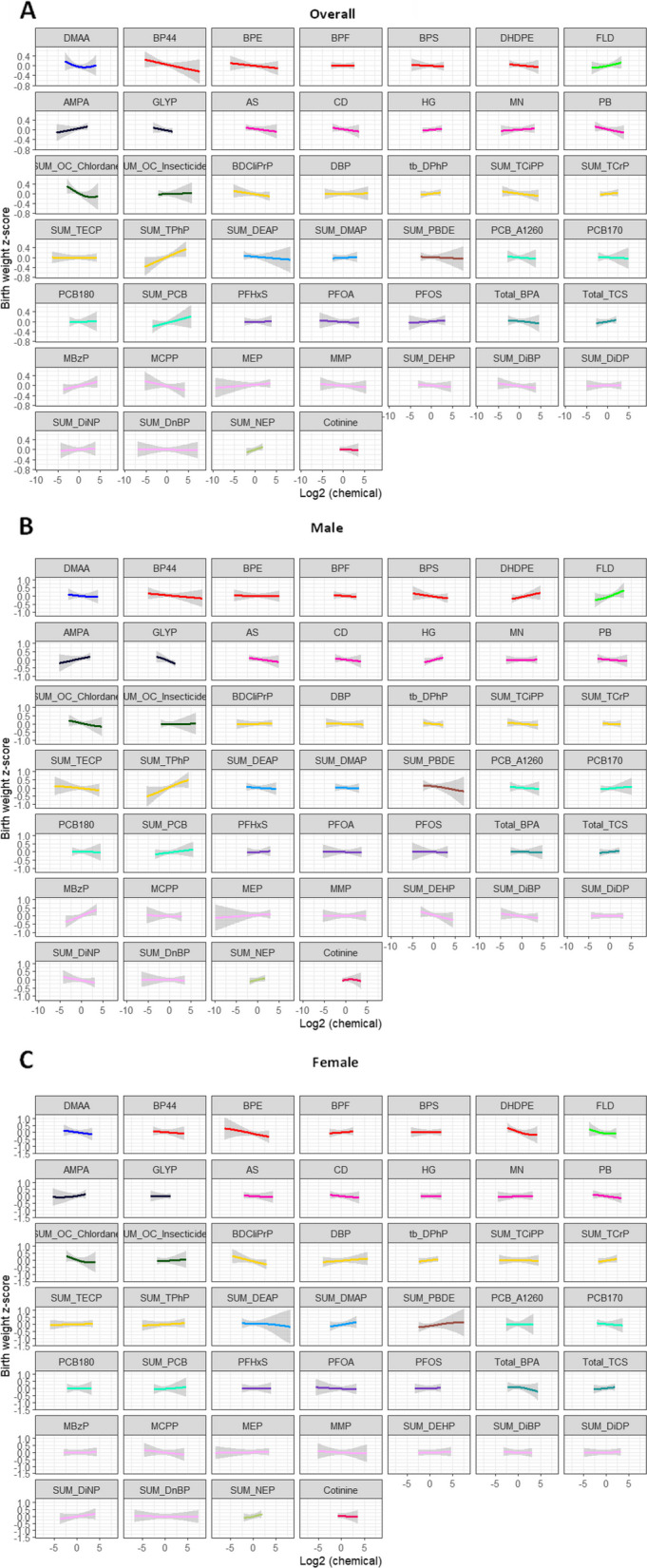
Univariate exposure–response function of each chemical exposure (95% credible intervals) with birth weight z-score, where the remaining exposures are fixed at their median values, using BKMR, MIREC Study. **A** Overall sample; **B** Male Infants; **C** Female infants

According to the BKMR results, the highest group PIPs, which indicate the important chemical classes for the full sample were the OCs, arsenic, solvents, PCBs and metals. The individual chemical PIPs were highest for ΣOC Chlordane, DMAA, ΣNEP, ΣPCB, Cd and Pb. (Table S3) A graph of the exposure–response function (95% CI) between chemical concentrations and birth weight z-score, while fixing the concentrations of other chemicals at their median values, and the covariates held constant, is shown in Fig. 3. This allowed us to visualise the relationships between each chemical and birth weight and assess non linearity. Most of the effect estimates contained the null, however the largest significant inverse association was observed for ΣOC Chlordane, with a difference in birth weight z-score of −0.121 (−0.235, −0.007) when going from its 25th to 75th percentile, and keeping all other exposures at their median (Table S3). Focusing on those with the highest PIPs, we see inverse relationships with birth weight z-score for Pb, Cd, DMAA and, while positive relationships were found for ΣPCB and ΣNEP.

The female only model PIPs indicated that DMAA, BPE, DHDPE, fluoride, glyphosate, Cd, Pb, ΣOC Chlordane, ΣPCB, and ΣNEP were the most important. The largest inverse association with birth weight z-score, of −0.148 (−0.314, 0.018) was for DHDPE. The PIPs from the male model indicated that glyphosate, fluoride, ΣOC Chlordane, ΣOC Insecticides, ΣTPhP, MBzP, MCPP and ΣDEHP were the most important, with the largest change in birth weight z-score of −0.057 (−0.126, 0.013) for glyphosate.

**Fig. 4:**
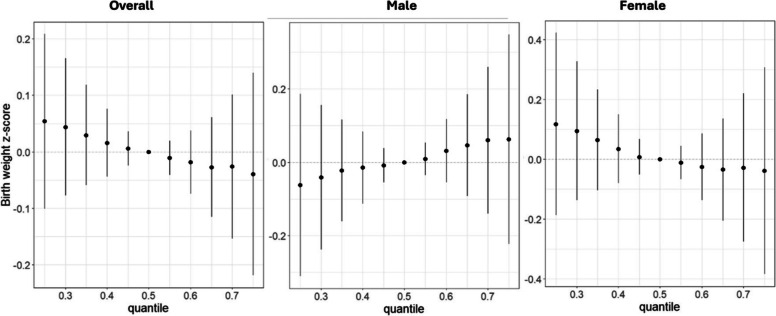
BKMR Overall risk (95% CI) of chemical exposures on birth weight z-score, when comparing all the chemicals at different percentiles with their median level, MIREC Study. Overall effect of the mixture (95% CI), defined as the difference in the response when all of the exposures are fixed at a specific quantile (ranging from 0.25 to 0.75), as compared to when all of the exposures are fixed at their median value. Models were adjusted for maternal education, parity, maternal country of birth, age and pre-pregnancy BMI

Another summary measure of interest using BKMR is to compute the overall effect of the chemicals, by comparing the value of the exposure–response function when all exposures are at a particular percentile as compared to when all of them are at their median values (Fig. 4)*.* For example, in the overall sample, the graph reveals that increasing levels of the joint exposure was associated with lower infant birth weight for the overall sample and especially for the female infants. The association between the joint exposure and birth weight z-score was positive in male infants.

**Fig. 5:**
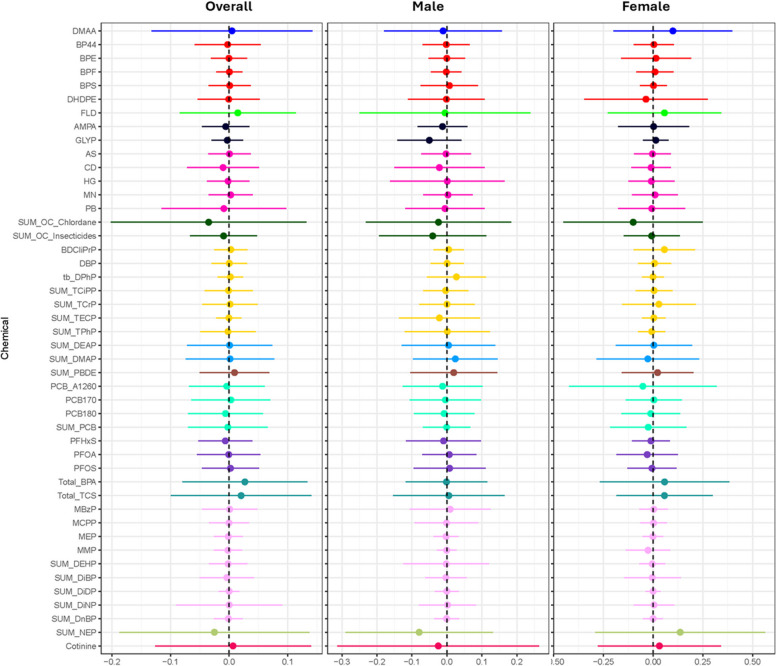
BKMR interactive effects, MIREC Study. Interaction is defined as the change in the single-exposure health effects when all of the remaining exposures are fixed at their 25th percentile as compared to when they are fixed at their 75th percentile. Models were adjusted for maternal education, parity, maternal country of birth, age and pre-pregnancy BMI

Interactive effects, defined as the change in the single-exposure health effects when all the remaining exposures are fixed at their 25th percentile as compared to when they are fixed at their 75th percentile are shown in Fig. 5*.* This plot shows no indication of interaction between the chemicals.

## Discussion

In this study, we considered the results of four different statistical methods to examine the associations between 46 chemicals and infant birth weight. PCA, k-means, WQSR and BKMR all allowed for the estimation of mixture health effects and the identification of important mixture components. From all biomarkers measured, DMAA, Pb, ΣOC Chlordane, ΣOC Insecticides, Aroclor 1260, PCB 170 and PCB 180 were most often associated with decreased birth weight, according to all four approaches, although the magnitude of the effect is quite small for most associations. Each approach identified some negative and positive associations between birth weight z-score and chemical biomarker concentrations. The statistically significant results varied between the methods; however, some findings across the methods were consistent. Chemicals identified as being significantly associated with birth weight z-score are summarized in Table 7. In addition to the mixture methods, we performed multiple linear regression on each individual chemical to compare to the multi-pollutant methods and there were many inverse associations with birth weight z-score. Although most of the associations were null, the results largely agreed with the findings from the mixture methods. Although our main focus was to compare the methods in terms of assessing the relationship between higher levels of chemical exposure with decreased birth weight, there were several positive associations found for some of the chemicals in our mixture.

**Table 7:** Summary comparison of significant positive and negative associations observed by method and chemical, MIREC Study

Chemical	**Multiple Linear Regression**		**Principal Component Analysis**		**K-Means Clustering**		**Weighted Quantile Sum Regression**		**Bayesian Kernal Machine Regression**	
	Male Infants	Female Infants	Male Infants	Female Infants	Male Infants	Female Infants	Male Infants	Female Infants	Male Infants	Female Infants
DMAA				**-**		**-**				
BP44				**-**		**-**				
BPE		**-**		**-**						
DHDPE		**-**		**-**		**-**				
As				**-**		**-**				
Cd				**-**						
Hg				**-**		**-**				
Pb				**-**		**-**				
BDCliPrP		**-**								
DBP				**-**						
ΣTCrP	**+ **									
ΣTPhP				**-**		**-**			**+ **	
ΣDEAP				**-**						
ΣOC Chlordane		**-**		**-**		**-**				
ΣOC Insecticides				**-**		**-**				
Aroclor 1260				**-**		**-**				
PCB 170				**-**		**-**				
PCB 180				**-**		**-**				
ΣPCB				**-**		**-**				
TCS	**+ **									
MBzP				**-**		**-**			**+ **	
MCPP				**-**		**-**				
ΣDEHP				**-**						
ΣDiBP				**-**		**-**				
ΣDiNP				**-**						
ΣDnBP				**-**		**-**				
Cotinine				**-**		**-**				

Our PCA analysis on the overall sample revealed an inverse association between principal components with large loadings for DMAA, Hg, Pb, ΣOC Chlordane, ΣOC Insecticides, ΣTPHP, PCBs, and several phthalates. Previously, a Flemish study of 152 mother-infant pairs utilized PCA methods to examine a mixture of 15 chemicals on birth weight. They found a significant association with a principal component comprised of PCB183, PCB153, and PCB180 [25]. Chui et al. found insignificant, moderate inverse relationships between principal components high in phthalate metabolites: MEHP, MEHHP, MEOHP and MECPP, MEP, MBP, MiBP, MBzP and birth weight [16]. Using a prospective cohort of 380 pregnant women, researchers studied 43 chemicals, and they found non-significant inverse associations with a principal component representing concentrations of ∑parabens, MEP, and Hg [34]. Another study, looking at phthalates and heavy metals, found that the principal component composed of MEHHP was moderately associated with a decrease in birth weight [41]. The PCA method is useful when the goal is to measure the effect of cumulative chemical exposures. It is very easy to use and efficient at producing components that are independent of one another to be used in further regression analysis. However, it is important to note that the components were derived in an unsupervised fashion by only reflecting the variability of the mixture and not taking into account the relationship with birth weight. Furthermore, interpreting the results is not straight forward, as the components are not on the same units as the original exposure variables and there is also no way to directly analyze non-linear associations or chemical interactions.

For the k-means clustering method we chose 4 clusters. The linear regression results for the overall sample indicated that the cluster inversely associated with birth weight z-score contained elevated concentrations of DMAA, As, Hg, Pb, ΣOC Chlordane, ΣOC Insecticides, Aroclor 1260, PCB170, PCB180 and ΣPCB. Some of these biomarker concentrations were also found by Kalloo et al., that looked at a mixture of 43 chemicals using k-means. They found the cluster associated with lower birth weight z-score and lower birth length, had high mean levels of most phenols, three phthalate metabolites, As, Hg, Cd, organophosphate and organochlorine pesticides, PCBs, and several PFAS [34]. The k-means algorithm is easy and efficient to implement and can be used in a wide variety of applications. Compared to hierarchical clustering methods, it may be less sensitive to outliers [54]. A limitation of k-means is that the number of clusters must be assigned beforehand and initial partitions can result in different final clusters. Cluster membership is determined by average chemical concentrations, making it impossible to gain insight into a dose response relationship.

From the WQS regression approach, the resulting index was shown to be inversely associated with birth weight z-score, when a inverse dissection was assumed. After running WQS repeated holdout validation, the results were attenuated towards the null. Results obtained without validation may not be incorrect; however, they may not generalize outside of the study and give overly optimistic results [55]. The highest weights were for Aroclor 1260, ΣOC Chlordane, GLYP, PCB180, MCPP, BP44, ΣDiBP, As, and Pb. In a previous study of metal exposures, WQSR analysis identified that copper (Cu), nickel (Ni), Mn, and Cd were weighted highly in a mixture index that was linked to decreased birthweight z-score [60]. In a study examining a mixture of pregnancy averaged phthalates, bisphenols, and organophosphate pesticides, using a quantile-based G-computation method [36], they found that infants in the fourth quartile of exposure had 91 g (95% CI: −258, 76) lower birth weight in comparison with those in the first quartile [56]. A primary advantage of the WQS regression approach is that it estimated empirical weights for the chemical contributions and does not produce transformations of variables. It has also been argued that restraining the effect in one direction may avoid reversal paradox, but it is important to consider both directions to provides separate representation of the relative importance of elements for overall positive or negative directional effects [14, 47]. WQSR has no way to directly analyze chemical interactions. Additionally, categorizing data into quantiles may result in a loss of robust data. WQS also poorly selects predictors in situations where the predictors are highly correlated to each other and weakly associated to the outcome of interest. To overcome this problem, we implemented repeated holdout validation, which allowed us to characterize weight uncertainty, aiding in the identification of toxic chemicals of concern [55].

The BKMR method revealed that increasing levels of the joint chemical biomarker concentrations were associated with lower infant birth weight z-scores, for the overall sample and for female infants. The joint association was positive in male infants. The chemicals with the largest PIPs and associated with decreased birth weight z-score for the whole sample were ΣOC Chlordane, Pb, Cd, DMAA, Aroclor 1260, and PCB170. There were chemicals with slightly lower PIPs that also displayed inverse associations with birth weight. In a previous study that looked at 8 phthalate metabolites, BKMR identified MEP and MEHP as having the strongest associations with decreased birth weight [16]. In two studies that looked at mixtures of metal elements and birth weight using BKMR, Hg and Ni were identified as the highest-ranking predictors of birthweight-for-gestational age [29, 30]. BKMR was also implemented in a study to measure the association between a mixture of 35 chemicals and birth weight, where they found that exposure to PCB180, PFAS congeners, PBDE 153, MEP, DMP and BPA displayed clear inverse associations with birth weight [61]. BKMR is especially useful to explore interactions between chemical exposures. This procedure is also useful when the exposure-outcome associations are non-linear. Variable selection and health effect estimation occurs simultaneously and provides an estimate of an overall mixture effect, and the importance of each exposure. The limitation of the method is the slow computation time. There are also no formal methods for evaluating the presence of interactions and non-linearity. Since we are looking at a high dimensional response surface, we must rely on visualization and there could be some difficulty with interpretation when comparing the results graphically. It should also be noted that choosing different priors may result in different PIPs.

Many previous studies reported sex-specific associations between birth outcomes and a number of environmental chemicals. Based on our methods, we more frequently found larger inverse relationships with birth weight and the chemical mixture in female infants, specifically for ΣOC Chlordane, BPE, DHDPE, PCB170, BDCLIPRP, BPA and PFOA. In contrast, male infants appeared to be more susceptible to ΣDEHP, ΣPBDE and glyphosate. A previous study using PCA, found that effects in girls were enhanced, with the highest association for mixtures containing thallium, PFOS, lead, cadmium, and manganese [25]. Our PCA results confirm some of these sex specific effects where we found that the effects of lead and cadmium were stronger in the female only models. In another study, using BKMR to assess metal mixtures, also found that for copper and selenium associations with birth weight were observed in female infants only [30]. In a population-based birth cohort study in Spain, Casas et al. showed that prenatal urinary MBzP concentrations were positively associated with birth weight among boys but not in girls [15]. Our BKMR results reveal a positive association between MBzP and birth weight z-score for the male infants only. While few studies have examined the impact of sexual dimorphism on placental growth, there has been evidence showing that the sex of the placenta influences fetal responses to external stimuli in utero and that females more readily respond to abrupt changes to intrauterine environment, which could lead to decreased fetal growth [48].

A major advantage of this research was the large sample size of 1127 participants, with suitable detection rates for 46 chemicals, making this cohort ideal to evaluate the efficacy of different mixture methods. Extensive sociodemographic and specimen collection factors were collected, that allowed us to control for a variety of covariates. Our study had a number of limitations. We used exploratory statistical approaches to discover profiles of chemicals that are predictive of birth weight, which resulted in a more complete understanding of the associations for this specific population, but generally provides little or no value for informing regulatory decisions [49]. Due to the complications of implementing the various methods on imputed data, our complete case analysis excluded 83 participants who were missing pre-pregnancy BMI. Individuals with a higher body weight may refrain from answering related questions, leading to a potential source of selection bias. Statistically speaking, the large number of chemicals in our mixture could increase the risk of false positive results and inadequate power to detect small effects could increase the risk of false negative results. Since we chose a minimal set of covariates, there is also potential for bias due to confounders that were not accounted for in the models. We created gestational age-specific birth weight z-scores as our outcome, since gestational age is on the causal pathway between chemical exposure and birth weight. Unfortunately, the interpretation of this metric is not straightforward as it reports effect measures in the unit of standard deviation, instead of grams. Additionally, we measured chemical biomarker concentrations using spot urine samples. Non-persistent chemicals such as phthalates, bisphenols, and OP pesticides have short elimination half-lives, thereby introducing measurement error and likely attenuating associations towards the null compared to associations for persistent chemicals. Furthermore, our cohort was on average from a higher socio-economic status than the general population of pregnant women in Canada [1] and therefore these results should not be generalized to other populations.

Importantly, the results emphasize the necessity of considering exposure to multiple chemicals simultaneously, as these substances can interact and exhibit combined effects on health outcomes. Recognizing the strengths and limitations of each approach can help inform which is the best method for their research question, and underscores the value of multi-method evaluations in environmental epidemiology. Future research should focus on advancing statistical methodologies to enhance our capacity to decipher the intricate relationships between environmental exposures and health outcomes. By clarifying inconsistent findings across various studies and integrating diverse methodological approaches, we can ultimately improve our understanding of the impact of chemicals on developmental health and inform public health policies aimed at reducing harmful exposures during critical periods of development.

In conclusion, this study significantly contributes to our understanding of the complex relationships between prenatal exposure to a mixture of environmental chemicals and birth weight outcomes in infants. Utilizing four distinct statistical methodologies—Principal Component Analysis (PCA), k-means clustering, Weighted Quantile Sum Regression (WQSR) and Bayesian Kernel Machine Regression (BKMR)—provided a comprehensive analysis of how these chemical exposures influence infant birth weight. Our findings indicate consistent inverse associations for certain chemicals across methods, particularly ΣOC Chlordane, which suggests that prenatal exposure to these compounds may adversely impact fetal growth, especially in female infants.

## Supplementary Information

Supplementary Material 1.

## Data Availability

The datasets generated and/or analysed during the current study are not publicly available to respect privacy, but individuals may apply to access the data through the MIREC Biobank (https://www.mirec-canada.ca/en).
