# Supplementary material for: Chemical mixtures and birth weight: comparison of multipollutant models in the Maternal-Infant Research on Environmental Chemicals (MIREC) study
Source: Environ Health. 2026 May 22;25:62. doi: 10.1186/s12940-026-01300-z (PMC13386715; doi:10.1186/s12940-026-01300-z)
Supplement: Supplementary file 1 — Supplementary Material 1. [file 12940_2026_1300_MOESM1_ESM.docx]

**Supplemental Material**

Chemical mixtures and birth weight: comparison of multipollutant models in the Maternal-Infant Research on Environmental Chemicals (MIREC) Study

**Table of contents:**

| **Figure S1.** Directed Acyclic Graph for first trimester chemicals and infant birth weight  **Table S1.** Distribution of first trimester EDC concentrations  **Table S2**. Distribution of first trimester EDC concentrations not included in our sample  **Figure S2**. Pearson correlation matrix for first trimester log-transformed maternal EDCs  **Figure S3.** The weights based on weighted quantile sum regression (WQSR) in the negative direction with birth weight z-score.  **Figure S4.** The weights based on weighted quantile sum regression (WQSR) in the positive direction with birth weight z-score.  **Table S3:** BKMR group and conditional Posterior Inclusion Probabilities (PIPs) and effect estimates (95% CIs) | p. 2    p. 2  p. 4  p. 6  p. 8  p. 10  p. 12 |
| --- | --- |

**Figure S1. Directed Acyclic Graph for trimester 1 EDCs and infant birthweight**
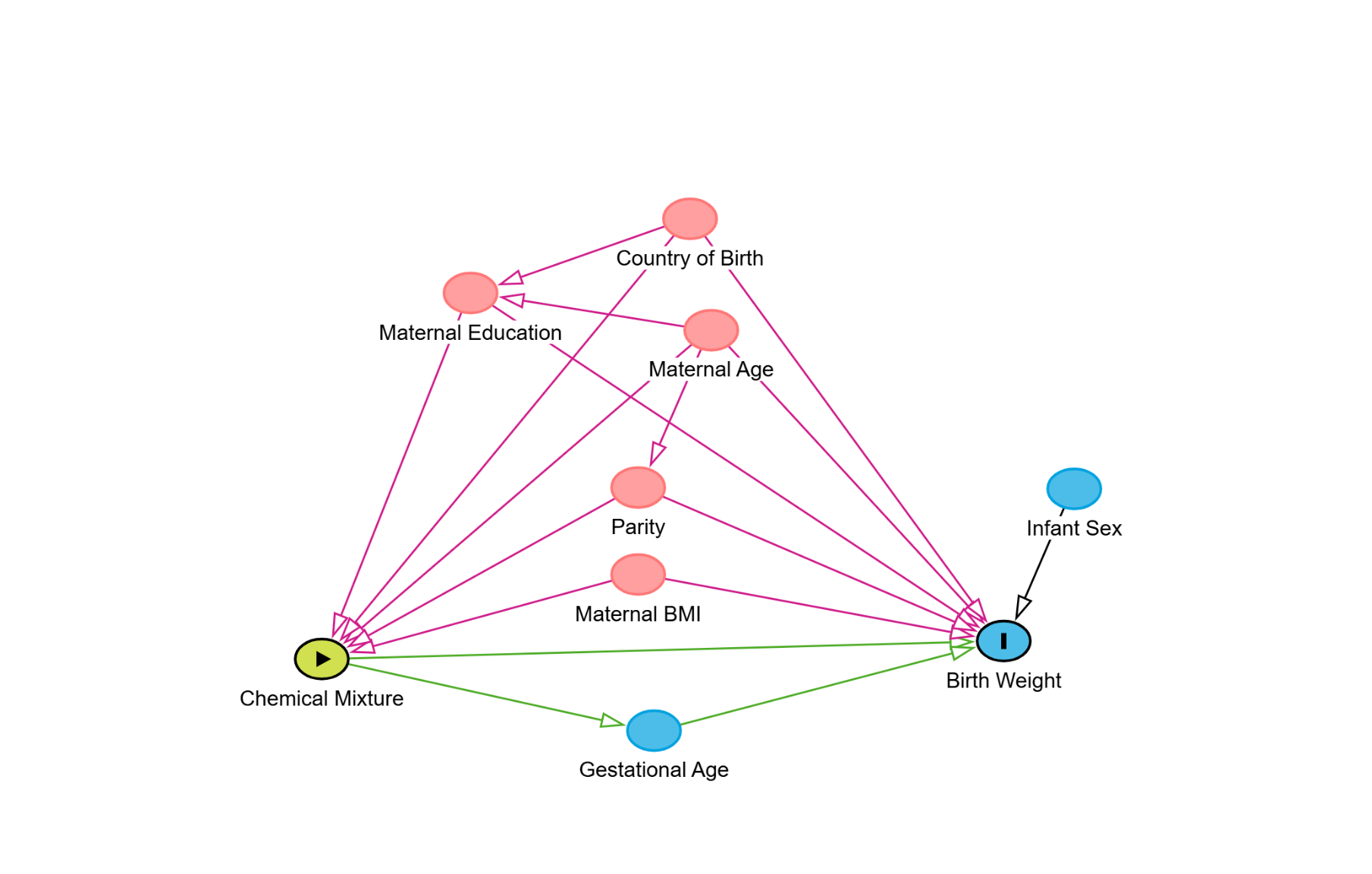


**Table S1. Distribution of first trimester EDC concentrations (N = 1127)**

| Chemical Group | Chemical ^1^ | Units | LOD | % > LOD | Min | 25th Percentile | 50th Percentile | 75th Percentile | Max | Geometric Mean (95% CI) |
| --- | --- | --- | --- | --- | --- | --- | --- | --- | --- | --- |
| Arsenic^3^ | **DMAA** | μg/L | 0.75 | 86.87 | <LOD | 1.669 | 2.411 | 3.942 | 44.14 | 2.69 (2.59, 2.8) |
| Bisphenol analogues^2^ | **BP44** | μg/L | 0.01 | 99.47 | <LOD | 0.206 | 0.295 | 0.451 | 93.79 | 0.32 (0.3, 0.33) |
| Bisphenol analogues^2^ | **BPE** | μg/L | 0.002 | 97.87 | <LOD | 0.015 | 0.023 | 0.037 | 4.70 | 0.02 (0.02, 0.03) |
| Bisphenol analogues^2^ | **BPF** | μg/L | 0.03 | 72.94 | <LOD | 0.025 | 0.076 | 0.211 | 112.84 | 0.05 (0.04, 0.06) |
| Bisphenol analogues^2^ | **BPS** | μg/L | 0.06 | 69.83 | <LOD | 0.059 | 0.117 | 0.266 | 18.61 | 0.11 (0.1, 0.12) |
| Bisphenol analogues^2^ | **DHDPE** | μg/L | 0.04 | 99.29 | 0.056 | 0.298 | 0.489 | 0.912 | 49.77 | 0.56 (0.53, 0.59) |
| Herbicides^2^ | **AMPA** | μg/L | 0.09 | 73.47 | <LOD | 0.112 | 0.210 | 0.385 | 6.14 | 0.16 (0.15, 0.18) |
| Herbicides^2^ | **GLYP** | μg/L | 0.08 | 75.07 | <LOD | 0.120 | 0.250 | 0.474 | 3.91 | 0.11 (0.1, 0.13) |
| Metals^3^ | **As** | μg/L | 0.22 | 92.28 | <LOD | 0.509 | 0.749 | 1.124 | 34.46 | 0.75 (0.72, 0.78) |
| Metals^3^ | **Cd** | μg/L | 0.04 | 97.60 | <LOD | 0.135 | 0.202 | 0.315 | 4.50 | 0.22 (0.21, 0.23) |
| Metals^3^ | **Hg** | μg/L | 0.12 | 89.88 | <LOD | 0.341 | 0.722 | 1.404 | 9.43 | 0.63 (0.6, 0.67) |
| Metals^3^ | **Mn** | μg/L | 0.55 | 100.00 | 2.143 | 7.143 | 8.791 | 10.989 | 28.57 | 8.77 (8.61, 8.94) |
| Metals^3^ | **Pb** | μg/dL | 0.1 | 100.00 | 0.155 | 0.435 | 0.622 | 0.850 | 4.14 | 0.62 (0.61, 0.64) |
| OrganoChlorines^3^ | **ΣOC Chlordane** | μg/kg lipids | NA | NA | 2.578 | 5.829 | 7.483 | 9.857 | 48.00 | 7.72 (7.55, 7.91) |
| OrganoChlorines^3^ | **ΣOC Insecticides** | μg/kg lipids | NA | NA | 18.424 | 50.663 | 67.663 | 97.640 | 5919.01 | 78.44 (75.23, 81.78) |
| OPFRs^2^ | **BDCliPrP** | µg/L | 0.05 | 89.26 | <LOD | 0.159 | 0.311 | 0.602 | 22.19 | 0.29 (0.27, 0.32) |
| OPFRs^2^ | **DBP** | µg/L | 0.05 | 69.03 | <LOD | <LOD | 0.073 | 0.129 | 22.72 | 0.08 (0.07, 0.08) |
| OPFRs^2^ | **tb_DPhP** | µg/L | 0.008 | 62.02 | <LOD | <LOD | 0.014 | 0.024 | 0.55 | 0.01 (0.01, 0.01) |
| OPFRs^2^ | **ΣTCiPP** | nmol/L | NA | NA | 0.011 | 0.855 | 1.598 | 3.398 | 567.83 | 1.77 (1.64, 1.9) |
| OPFRs^2^ | **ΣTCrP** | nmol/L | NA | NA | 0.000 | 0.001 | 0.013 | 0.041 | 1.26 | 0.01 (0.01, 0.01) |
| OPFRs^2^ | **ΣTECP** | nmol/L | NA | NA | 0.001 | 0.654 | 1.170 | 2.475 | 166.74 | 1.31 (1.23, 1.4) |
| OPFRs^2^ | **ΣTPhP** | nmol/L | NA | NA | 0.006 | 1.556 | 2.684 | 4.939 | 596.81 | 2.78 (2.6, 2.98) |
| OPs^3^ | **ΣDEAP** | nmol/L | NA | NA | 3.627 | 15.111 | 24.125 | 39.658 | 13666.76 | 25.08 (24.01, 26.21) |
| OPs^3^ | **ΣDMAP** | nmol/L | NA | NA | 5.168 | 32.300 | 63.674 | 123.316 | 1863.73 | 64 (60.37, 67.85) |
| PBDEs^3^ | **ΣPBDE** | μg/kg lipids | NA | NA | 12.602 | 31.460 | 38.082 | 50.519 | 1780.36 | 42.62 (41.43, 43.85) |
| PCBs^3^ | **Aroclor 1260** | μg/kg lipids | 0.1 | 98.14 | 8.951 | 38.710 | 60.000 | 95.313 | 1183.33 | 63.71 (61.08, 66.46) |
| PCBs^3^ | **PCB170** | μg/kg lipids | 0.01 | 55.72 | 0.442 | 1.219 | 1.725 | 3.016 | 71.67 | 2.08 (2, 2.17) |
| PCBs^3^ | **PCB180** | μg/kg lipids | 0.01 | 93.70 | 0.842 | 3.036 | 4.918 | 8.148 | 183.33 | 5.21 (4.97, 5.47) |
| PCBs^3^ | **ΣPCB** | μg/kg lipids | NA | NA | 1.768 | 5.143 | 6.110 | 7.678 | 58.28 | 6.57 (6.42, 6.71) |
| PFAS^3^ | **PFHxS** | μg/L | 0.22 | 96.01 | <LOD | 0.660 | 1.000 | 1.600 | 25.00 | 1.05 (1, 1.1) |
| PFAS^3^ | **PFOA** | μg/L | 0.1 | 99.82 | <LOD | 1.200 | 1.700 | 2.500 | 16.00 | 1.7 (1.65, 1.76) |
| PFAS^3^ | **PFOS** | μg/L | 0.34 | 99.91 | <LOD | <LOD | 4.700 | 6.800 | 32.00 | 4.58 (4.43, 4.74) |
| Phenols^3^ | **BPA** | μg/L | 0.47 | 88.20 | <LOD | 0.494 | 0.884 | 1.473 | 79.13 | 0.95 (0.9, 1.01) |
| Phenols^2^ | **TCS** | μg/L | 0.12 | 99.47 | <LOD | 2.586 | 9.670 | 114.800 | 1980.43 | 15.33 (13.48, 17.44) |
| Phthalates^2^ | **MBzP** | µg/L | 0.1, 0.4 | 95.39 | <LOD | 1.579 | 3.110 | 6.860 | 476.97 | 3.24 (3.03, 3.46) |
| Phthalates^2^ | **MCPP** | µg/L | 0.1 | 87.84 | <LOD | 0.413 | 0.714 | 1.280 | 159.99 | 0.72 (0.67, 0.78) |
| Phthalates^2^ | **MEP** | µg/L | 0.8, 1.0 | 99.02 | <LOD | 11.562 | 25.079 | 65.864 | 8599.57 | 29.3 (26.76, 32.09) |
| Phthalates^2^ | **MMP** | µg/L | 0.2 | 96.18 | <LOD | 1.193 | 1.780 | 2.765 | 424.88 | 1.73 (1.63, 1.83) |
| Phthalates^2^ | **ΣDEHP** | nmol/L | NA | NA | 4.073 | 40.765 | 69.093 | 115.135 | 5861.48 | 72.74 (68.89, 76.8) |
| Phthalates^2^ | **ΣDiBP** | nmol/L | NA | NA | 0.621 | 24.407 | 41.855 | 69.007 | 1043.50 | 40.8 (38.92, 42.78) |
| Phthalates^2^ | **ΣDiDP** | nmol/L | NA | NA | 0.002 | 1.594 | 2.696 | 5.035 | 353.41 | 2.46 (2.26, 2.69) |
| Phthalates^2^ | **ΣDiNP** | nmol/L | NA | NA | 0.019 | 4.252 | 8.409 | 21.553 | 3651.57 | 9.72 (8.92, 10.59) |
| Phthalates^2^ | **ΣDnBP** | nmol/L | NA | NA | 0.021 | 23.464 | 45.215 | 78.118 | 146505.67 | 42.9 (40.21, 45.77) |
| Solvents^2^ | **ΣNEP** | µmol/L | NA | NA | 0.005 | 1.177 | 13.005 | 288.946 | 12588.17 | 11.24 (8.97, 14.07) |
| Tobacco Metabolite^3^ | **Cotinine** | ng/ml | 0.37 | 53.06 | <LOD | <LOD | <LOD | 0.200 | 290.00 | 0.03 (0.02, 0.03) |
| Flouride^2^ | **FLD** | µg/mL | 0.02 | NA | <LOD | 0.183 | 0.338 | 0.565 | 7.25 | 0.3 (0.28, 0.32) |

^1^ The full chemical names are in Table 1.

^2^ Machine readings were available for results below the limit of detection.

^3^ Results below the limit of detection (LOD) were replaced by LOD/√2.

Urinary chemical concentrations were specific gravity standardized. PolyBrominated Diphenyl Ethers (PBDEs), PolyChlorinated Biphenyls (PCBs) and OrganoChlorine compounds (OCs) were lipid standardized.

**Table S2. Distribution of first trimester EDC concentrations not included in our sample (N = 733)**

| Chemical Group | Chemical ^1^ | Units | n | LOD | % > LOD | Min | 25th Percentile | 50th Percentile | 75th Percentile | Max | Geometric Mean (95% CI) |
| --- | --- | --- | --- | --- | --- | --- | --- | --- | --- | --- | --- |
| **Arsenic^3^** | **DMAA** | μg/L | 684 | 0.75 | 83.48 | <LOD | 1.62 | 2.40 | 3.61 | 38.07 | 2.69 (2.59, 2.8) |
| **Bisphenol analogues^2^** | **BP44** | μg/L | 603 | 0.01 | 100.00 | 0.05 | 0.19 | 0.29 | 0.47 | 26.57 | 0.32 (0.3, 0.33) |
| **Bisphenol analogues^2^** | **BPE** | μg/L | 609 | 0.002 | 96.55 | <LOD | 0.01 | 0.02 | 0.04 | 0.98 | 0.02 (0.02, 0.03) |
| **Bisphenol analogues^2^** | **BPF** | μg/L | 606 | 0.03 | 69.47 | <LOD | <LOD | 0.08 | 0.25 | 806.91 | 0.05 (0.04, 0.06) |
| **Bisphenol analogues^2^** | **BPS** | μg/L | 585 | 0.06 | 65.81 | <LOD | 0.06 | 0.11 | 0.24 | 9.71 | 0.11 (0.1, 0.12) |
| **Bisphenol analogues^2^** | **DHDPE** | μg/L | 593 | 0.04 | 97.13 | 0.04 | 0.28 | 0.51 | 1.00 | 261.44 | 0.56 (0.53, 0.59) |
| **Herbicides^2^** | **AMPA** | μg/L | 605 | 0.09 | 68.43 | <LOD | 0.11 | 0.21 | 0.38 | 2.58 | 0.16 (0.15, 0.18) |
| **Herbicides^2^** | **GLYP** | μg/L | 587 | 0.08 | 70.70 | <LOD | 0.11 | 0.24 | 0.44 | 3.41 | 0.11 (0.1, 0.13) |
| **Metals^3^** | **As** | μg/L | 694 | 0.22 | 93.37 | <LOD | 0.52 | 0.82 | 1.20 | 14.23 | 0.75 (0.72, 0.78) |
| **Metals^3^** | **Cd** | μg/L | 694 | 0.04 | 96.69 | <LOD | 0.13 | 0.20 | 0.31 | 5.06 | 0.22 (0.21, 0.23) |
| **Metals^3^** | **Hg** | μg/L | 694 | 0.12 | 91.50 | <LOD | 0.30 | 0.66 | 1.26 | 10.03 | 0.63 (0.6, 0.67) |
| **Metals^3^** | **Mn** | μg/L | 694 | 0.55 | 100.00 | 2.03 | 7.14 | 8.79 | 10.99 | 29.12 | 8.77 (8.61, 8.94) |
| **Metals^3^** | **Pb** | μg/dL | 694 | 0.1 | 100.00 | 0.18 | 0.44 | 0.60 | 0.83 | 3.52 | 0.62 (0.61, 0.64) |
| **OrganoChlorines^3^** | **ΣOC Chlordane** | μg/kg lipids | 689 | NA | NA | 2.25 | 5.47 | 7.12 | 9.23 | 27.93 | 7.21 (7.01, 7.42) |
| **OrganoChlorines^3^** | **ΣOC Insecticides** | μg/kg lipids | 689 | NA | NA | 20.90 | 46.20 | 62.86 | 91.58 | 2751.48 | 73.91 (69.86, 78.19) |
| **OPFRs^2^** | **BDCliPrP** | µg/L | 579 | 0.05 | 88.08 | <LOD | 0.16 | 0.32 | 0.65 | 19.40 | 0.31 (0.28, 0.34) |
| **OPFRs^2^** | **DBP** | µg/L | 608 | 0.05 | 65.63 | <LOD | 0.05 | 0.07 | 0.12 | 2.40 | 0.08 (0.07, 0.08) |
| **OPFRs^2^** | **tb_DPhP** | µg/L | 526 | 0.008 | 51.14 | <LOD | <LOD | 0.01 | 0.02 | 0.48 | 0.01 (0, 0.01) |
| **OPFRs^2^** | **ΣTCiPP** | nmol/L | 621 | NA | NA | 0.05 | 0.78 | 1.58 | 3.72 | 240.57 | 1.75 (1.58, 1.94) |
| **OPFRs^2^** | **ΣTCrP** | nmol/L | 621 | NA | NA | 0.00 | 0.00 | 0.01 | 0.04 | 2.10 | 0.01 (0.01, 0.01) |
| **OPFRs^2^** | **ΣTECP** | nmol/L | 621 | NA | NA | 0.00 | 0.66 | 1.25 | 2.61 | 134.35 | 1.27 (1.14, 1.41) |
| **OPFRs^2^** | **ΣTPhP** | nmol/L | 621 | NA | NA | 0.00 | 1.47 | 2.60 | 4.99 | 116.71 | 2.61 (2.37, 2.86) |
| **OPs^3^** | **ΣDEAP** | nmol/L | 684 | NA | NA | 4.32 | 15.11 | 23.19 | 36.33 | 292.05 | 24.24 (22.95, 25.6) |
| **OPs^3^** | **ΣDMAP** | nmol/L | 684 | NA | NA | 4.04 | 27.98 | 59.02 | 128.14 | 1309.95 | 61.2 (56.53, 66.26) |
| **PBDEs^3^** | **ΣPBDE** | μg/kg lipids | 682 | NA | NA | 17.69 | 31.39 | 37.70 | 49.28 | 571.78 | 42.05 (40.6, 43.56) |
| **PCBs^3^** | **Aroclor 1260** | μg/kg lipids | 689 | 0.1 | 96.08 | 8.62 | 34.62 | 53.97 | 85.07 | 655.17 | 55.11 (52.22, 58.15) |
| **PCBs^3^** | **PCB170** | μg/kg lipids | 689 | 0.01 | 50.07 | 0.59 | 1.14 | 1.57 | 2.68 | 40.30 | 1.88 (1.79, 1.97) |
| **PCBs^3^** | **PCB180** | μg/kg lipids | 689 | 0.01 | 90.28 | 0.59 | 2.68 | 4.38 | 7.19 | 114.93 | 4.46 (4.21, 4.73) |
| **PCBs^3^** | **ΣPCB** | μg/kg lipids | 689 | NA | NA | 2.36 | 4.96 | 5.86 | 7.19 | 47.69 | 6.18 (6.02, 6.35) |
| **PFAS^3^** | **PFHxS** | μg/L | 695 | 0.22 | 95.97 | <LOD | 0.64 | 1.00 | 1.50 | 40.00 | 1.01 (0.95, 1.07) |
| **PFAS^3^** | **PFOA** | μg/L | 695 | 0.1 | 99.86 | <LOD | 1.10 | 1.60 | 2.30 | 11.00 | 1.57 (1.5, 1.64) |
| **PFAS^3^** | **PFOS** | μg/L | 695 | 0.34 | 99.71 | <LOD | 3.20 | 4.60 | 6.80 | 36.00 | 4.53 (4.34, 4.74) |
| **Phenols^3^** | **BPA** | μg/L | 684 | 0.47 | 86.40 | <LOD | 0.50 | 0.82 | 1.51 | 204.29 | 0.92 (0.86, 0.99) |
| **Phenols^2^** | **TCS** | μg/L | 618 | 0.12 | 99.19 | <LOD | 2.52 | 7.90 | 67.63 | 4199.80 | 12.47 (10.55, 14.74) |
| **Phthalates^2^** | **MBzP** | µg/L | 599 | 0.1, 0.4 | 95.33 | <LOD | 1.46 | 2.96 | 6.61 | 238.21 | 3.2 (2.92, 3.52) |
| **Phthalates^2^** | **MCPP** | µg/L | 536 | 0.1 | 84.89 | <LOD | 0.41 | 0.74 | 1.30 | 60.86 | 0.7 (0.62, 0.79) |
| **Phthalates^2^** | **MEP** | µg/L | 629 | 0.8, 1.0 | 98.89 | 0.47 | 10.28 | 22.82 | 62.88 | 3545.22 | 27.6 (24.79, 30.72) |
| **Phthalates^2^** | **MMP** | µg/L | 551 | 0.2 | 94.19 | <LOD | 1.21 | 1.85 | 2.93 | 46.84 | 1.65 (1.5, 1.81) |
| **Phthalates^2^** | **ΣDEHP** | nmol/L | 635 | NA | NA | 5.82 | 37.01 | 62.25 | 104.13 | 3920.09 | 67.13 (62.58, 72) |
| **Phthalates^2^** | **ΣDiBP** | nmol/L | 633 | NA | NA | 2.45 | 22.51 | 38.78 | 66.69 | 1117.90 | 39.13 (36.63, 41.8) |
| **Phthalates^2^** | **ΣDiDP** | nmol/L | 635 | NA | NA | 0.00 | 1.63 | 2.73 | 4.83 | 1173.63 | 2.33 (2.05, 2.63) |
| **Phthalates^2^** | **ΣDiNP** | nmol/L | 635 | NA | NA | 0.05 | 3.60 | 8.10 | 16.90 | 1826.32 | 8.61 (7.7, 9.62) |
| **Phthalates^2^** | **ΣDnBP** | nmol/L | 626 | NA | NA | 0.03 | 22.54 | 42.05 | 77.90 | 3775.64 | 39.84 (36.55, 43.42) |
| **Solvents^2^** | **ΣNEP** | µmol/L | 621 | NA | NA | 0.00 | 0.24 | 9.45 | 350.92 | 17243.17 | 7.71 (5.56, 10.68) |
| **Tobacco Metabolite^3^** | **Cotinine** | ng/ml | 695 | 0.37 | 55.40 | <LOD | <LOD | <LOD | <LOD | 290.00 | 0.03 (0.03, 0.04) |
| **Flouride^2^** | **FLD** | µg/mL | 630 | 0.02 | 100.00 | <LOD | 0.17 | 0.32 | 0.57 | 4.64 | 0.31 (0.28, 0.33) |

^1^ The full chemical names are in Table 1.

^2^ Machine readings were available for results below the limit of detection.

^3^ Results below the limit of detection (LOD) were replaced by LOD/√2.

Urinary chemical concentrations were specific gravity standardized. PolyBrominated Diphenyl Ethers (PBDEs), PolyChlorinated Biphenyls (PCBs) and OrganoChlorine compounds (OCs) were lipid standardized.

**Figure S2. Pearson correlation matrix for first trimester log-transformed maternal EDCs**
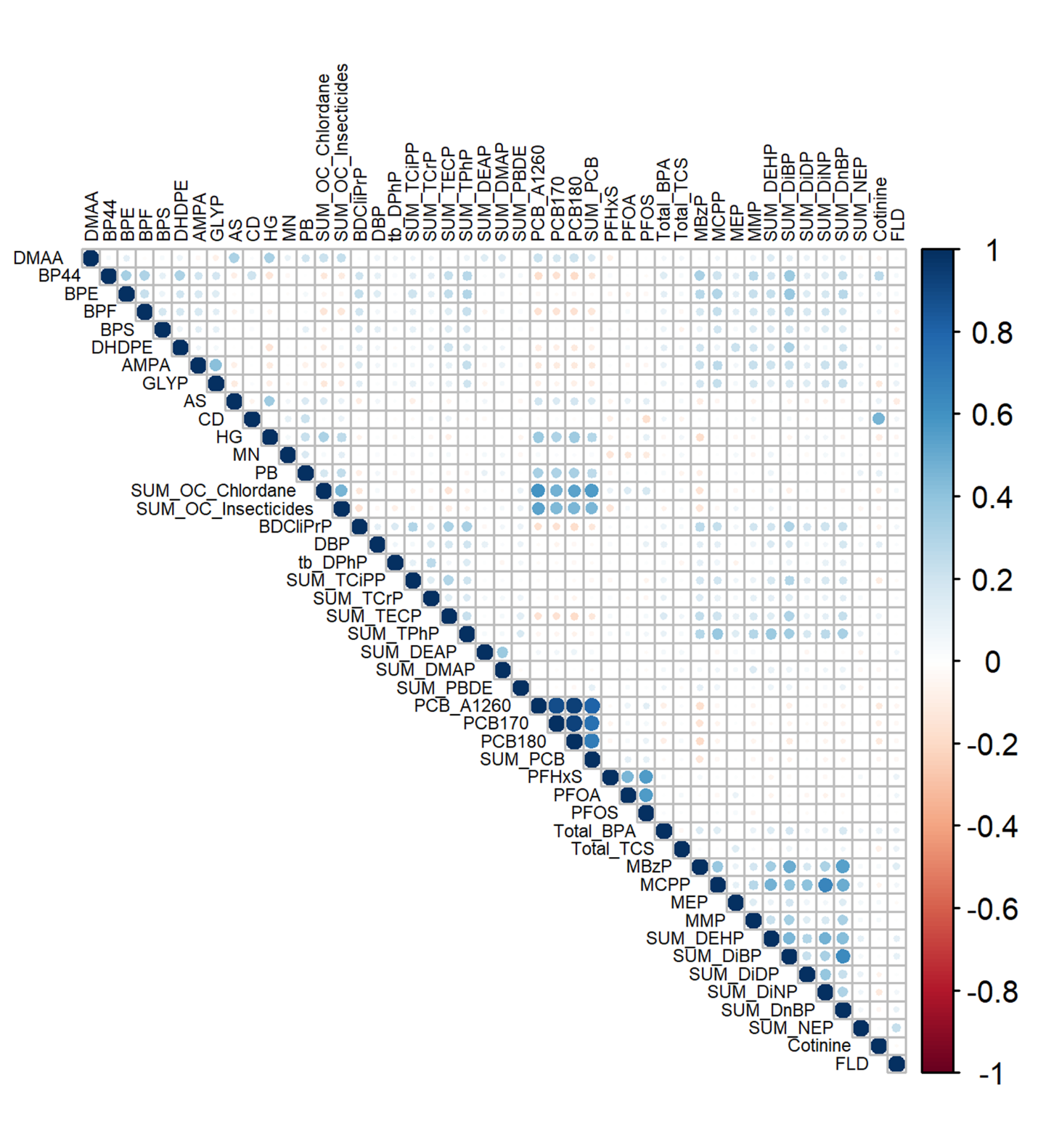


**Figure S3. The weights based on weighted quantile sum regression (WQSR) in the negative direction with birth weight z-score. A: Overall sample; B: Male Infants; C: Female infants**


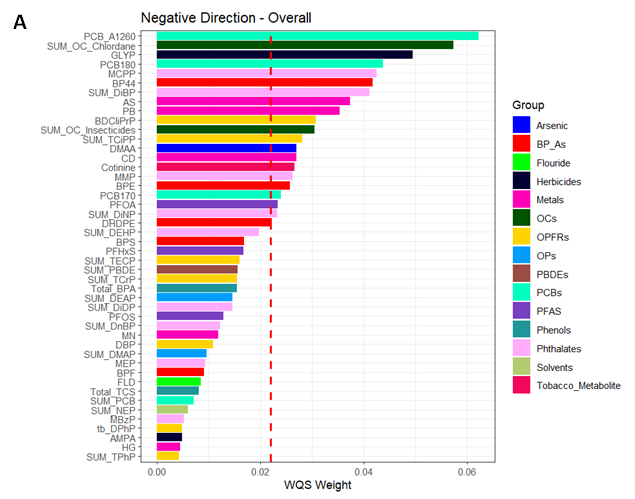


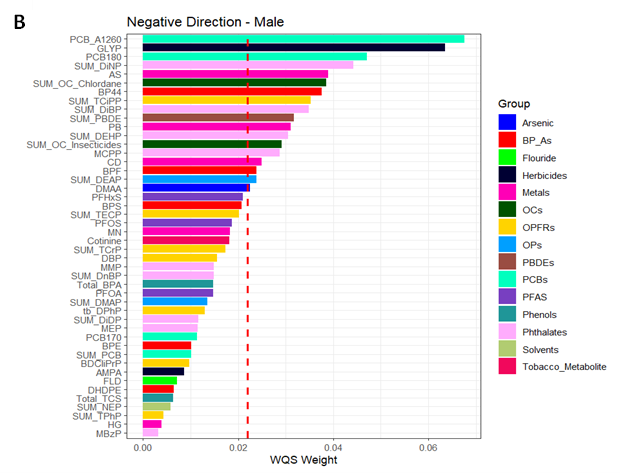


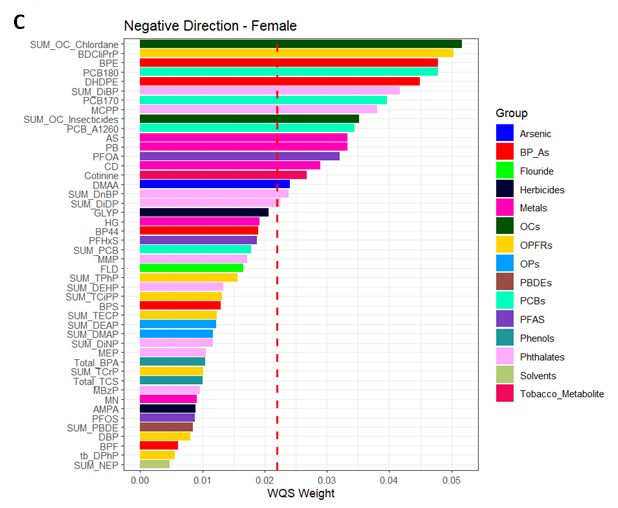


**Figure S4. The weights based on weighted quantile sum regression (WQSR) in the positive direction with birth weight z-score. A: Overall sample; B: Male Infants; C: Female infants**


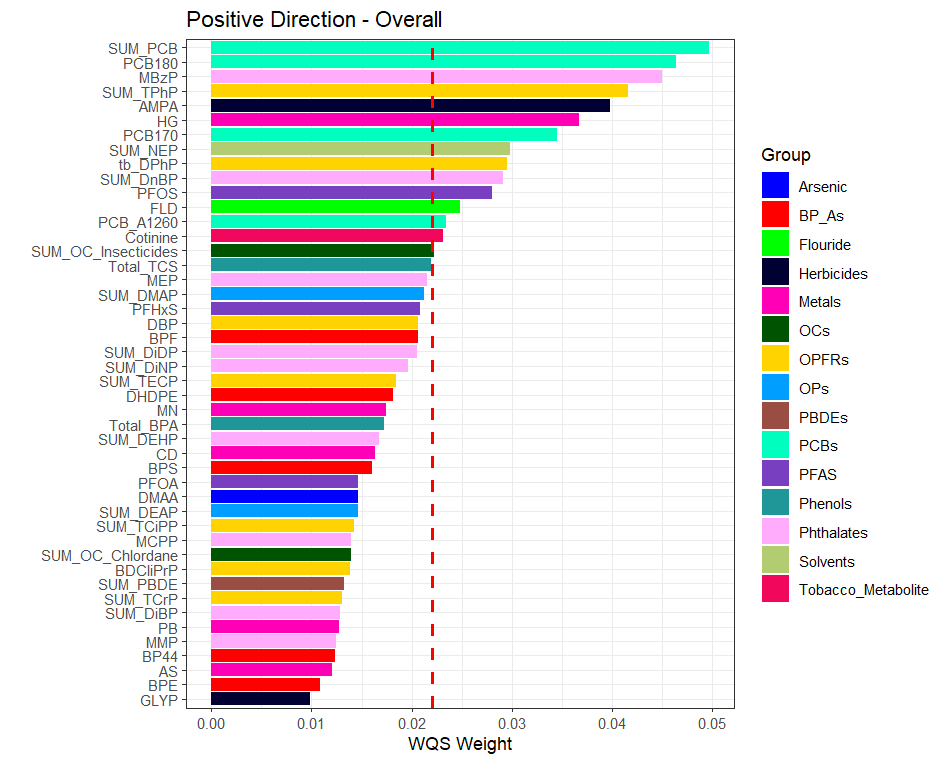


**A**


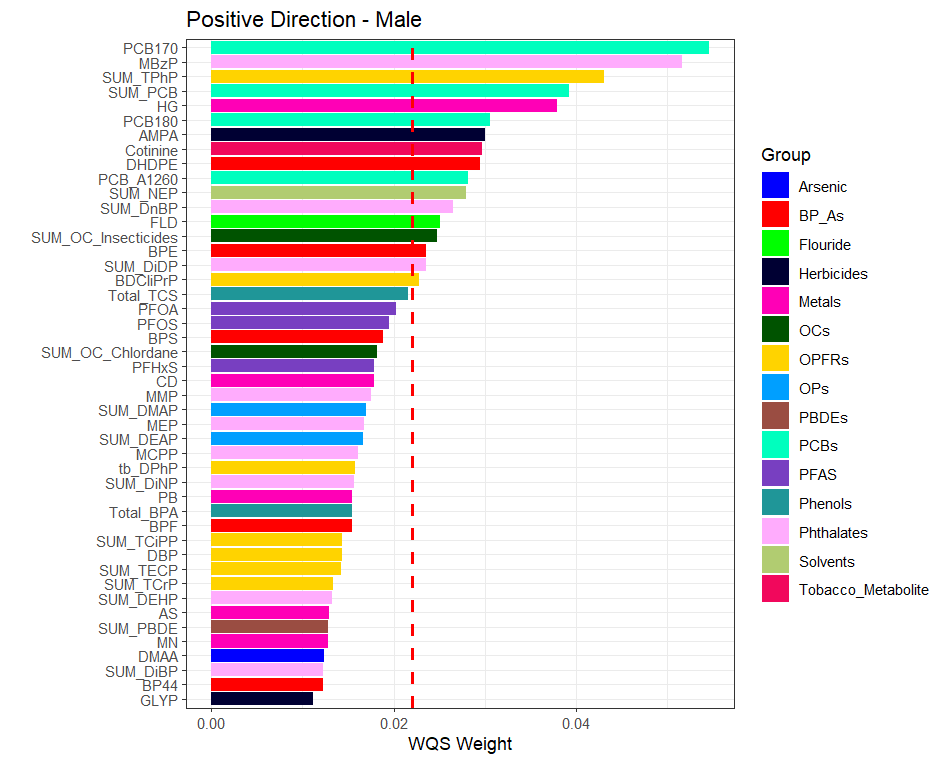


**B**


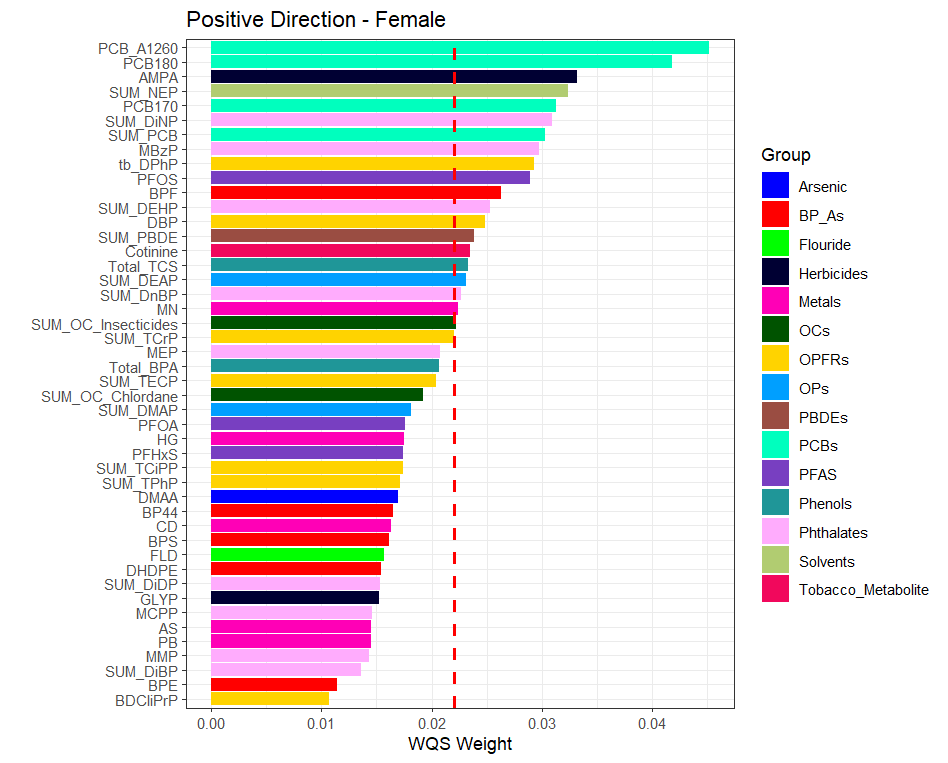


**C**

**Table S3. BKMR group and conditional Posterior Inclusion Probabilities (PIPs) and effect estimates (95% CIs)**

|  |  | OVERALL (n=1127) | | | MALES (n =589) | | | FEMALES (n = 538) | | |
| --- | --- | --- | --- | --- | --- | --- | --- | --- | --- | --- |
| Chemical Group | **Chemical** | **Group PIP** | **Cond. PIP** | **Estimate^1^ (95% CI)** | **Group PIP** | **Cond. PIP** | **Estimate^1^ (95% CI)** | **Group PIP** | **Cond. PIP** | **Estimate^1^ (95% CI)** |
| Arsenic | DMAA | **0.206** | **1.000** | -0.038 (-0.128, 0.053) | 0.190 | 1.000 | -0.029 (-0.156, 0.099) | **0.321** | **1.000** | -0.064 (-0.224, 0.095) |
| Bisphenol analogues | BP44 | 0.141 | 0.323 | -0.046 (-0.115, 0.024) | 0.221 | 0.174 | -0.037 (-0.124, 0.05) | **0.447** | 0.117 | -0.02 (-0.117, 0.078) |
| Bisphenol analogues | BPE | 0.141 | 0.159 | -0.017 (-0.066, 0.031) | 0.221 | 0.127 | -0.005 (-0.071, 0.061) | **0.447** | **0.295** | -0.07 (-0.186, 0.046) |
| Bisphenol analogues | BPF | 0.141 | 0.106 | 0 (-0.036, 0.036) | 0.221 | 0.128 | -0.012 (-0.064, 0.04) | **0.447** | 0.108 | 0.021 (-0.056, 0.098) |
| Bisphenol analogues | BPS | 0.141 | 0.180 | -0.007 (-0.055, 0.041) | 0.221 | 0.358 | -0.032 (-0.112, 0.047) | **0.447** | 0.050 | 0 (-0.065, 0.066) |
| Bisphenol analogues | DHDPE | 0.141 | 0.232 | -0.017 (-0.083, 0.049) | 0.221 | 0.212 | 0.058 (-0.041, 0.157) | **0.447** | **0.430** | -0.148 (-0.314, 0.018) |
| Fluoride | FLD | 0.130 | 1.000 | 0.038 (-0.04, 0.116) | **0.357** | **1.000** | 0.124 (-0.014, 0.262) | **0.285** | **1.000** | -0.036 (-0.184, 0.111) |
| Herbicides | AMPA | 0.104 | 0.544 | 0.027 (-0.02, 0.073) | **0.284** | 0.301 | 0.036 (-0.033, 0.106) | 0.235 | 0.582 | 0.045 (-0.076, 0.165) |
| Herbicides | GLYP | 0.104 | 0.456 | -0.017 (-0.049, 0.014) | **0.284** | **0.699** | -0.057 (-0.126, 0.013) | 0.235 | 0.418 | -0.002 (-0.058, 0.054) |
| Metals | As | **0.173** | 0.110 | -0.024 (-0.08, 0.031) | 0.217 | 0.225 | -0.041 (-0.123, 0.041) | **0.271** | 0.141 | -0.016 (-0.11, 0.077) |
| Metals | Cd | **0.173** | **0.354** | -0.027 (-0.097, 0.043) | 0.217 | 0.245 | -0.038 (-0.155, 0.078) | **0.271** | **0.219** | -0.026 (-0.114, 0.063) |
| Metals | Hg | **0.173** | 0.068 | 0.027 (-0.036, 0.09) | 0.217 | 0.186 | 0.099 (-0.036, 0.235) | **0.271** | 0.160 | -0.001 (-0.116, 0.114) |
| Metals | Mn | **0.173** | 0.078 | 0.015 (-0.046, 0.075) | 0.217 | 0.135 | 0.006 (-0.082, 0.095) | **0.271** | 0.178 | 0.013 (-0.087, 0.113) |
| Metals | Pb | **0.173** | **0.390** | -0.053 (-0.144, 0.039) | 0.217 | 0.209 | -0.026 (-0.143, 0.09) | **0.271** | **0.302** | -0.066 (-0.199, 0.067) |
| OrganoChlorines | ΣOC Chlordane | **0.406** | **0.838** | **-0.121 (-0.235, -0.007)** | **0.254** | **0.552** | -0.082 (-0.243, 0.079) | **0.413** | **0.748** | -0.147 (-0.354, 0.06) |
| OrganoChlorines | ΣOC Insecticides | **0.406** | 0.162 | 0.005 (-0.06, 0.071) | **0.254** | **0.448** | -0.01 (-0.132, 0.111) | **0.413** | 0.252 | 0.013 (-0.105, 0.132) |
| OPFRs | BDCliPrP | 0.113 | 0.137 | -0.023 (-0.072, 0.027) | **0.272** | 0.058 | 0.008 (-0.055, 0.072) | 0.298 | 0.353 | -0.095 (-0.212, 0.021) |
| OPFRs | DBP | 0.113 | 0.135 | 0.003 (-0.048, 0.053) | **0.272** | 0.067 | -0.009 (-0.076, 0.058) | 0.298 | 0.109 | 0.03 (-0.052, 0.111) |
| OPFRs | tb_DPhP | 0.113 | 0.150 | 0.012 (-0.02, 0.043) | **0.272** | 0.120 | -0.012 (-0.072, 0.047) | 0.298 | 0.126 | 0.025 (-0.027, 0.077) |
| OPFRs | ΣTCiPP | 0.113 | 0.167 | -0.023 (-0.082, 0.035) | **0.272** | **0.128** | -0.024 (-0.105, 0.057) | 0.298 | 0.088 | -0.01 (-0.098, 0.079) |
| OPFRs | ΣTCrP | 0.113 | 0.099 | 0.022 (-0.058, 0.101) | **0.272** | 0.076 | -0.005 (-0.115, 0.104) | 0.298 | 0.158 | 0.084 (-0.072, 0.24) |
| OPFRs | ΣTECP | 0.113 | 0.043 | -0.002 (-0.044, 0.041) | **0.272** | **0.142** | -0.036 (-0.146, 0.074) | 0.298 | 0.077 | 0.013 (-0.053, 0.079) |
| OPFRs | ΣTPhP | 0.113 | 0.269 | 0.067 (0.006, 0.129) | **0.272** | **0.408** | **0.123 (0.016, 0.23)** | 0.298 | 0.088 | 0.02 (-0.054, 0.094) |
| OPs | ΣDEAP | 0.097 | 0.486 | -0.02 (-0.098, 0.057) | 0.170 | 0.461 | -0.029 (-0.149, 0.09) | 0.222 | 0.451 | -0.025 (-0.159, 0.11) |
| OPs | ΣDMAP | 0.097 | 0.514 | 0.011 (-0.069, 0.092) | 0.170 | 0.539 | -0.011 (-0.125, 0.104) | 0.222 | 0.549 | 0.08 (-0.078, 0.238) |
| PBDEs | ΣPBDE | 0.085 | 1.000 | -0.007 (-0.066, 0.053) | 0.168 | 1.000 | -0.031 (-0.128, 0.066) | 0.221 | 1.000 | 0.048 (-0.061, 0.158) |
| PCBs | Aroclor 1260 | **0.170** | **0.245** | -0.023 (-0.145, 0.1) | 0.203 | 0.295 | -0.038 (-0.198, 0.121) | **0.303** | 0.214 | 0.002 (-0.295, 0.299) |
| PCBs | PCB170 | **0.170** | **0.233** | -0.013 (-0.13, 0.104) | 0.203 | 0.220 | 0.038 (-0.104, 0.179) | **0.303** | **0.254** | -0.031 (-0.192, 0.129) |
| PCBs | PCB180 | **0.170** | **0.245** | 0.006 (-0.11, 0.122) | 0.203 | 0.267 | -0.002 (-0.13, 0.126) | **0.303** | **0.224** | 0 (-0.172, 0.173) |
| PCBs | ΣPCB | **0.170** | **0.277** | 0.055 (-0.036, 0.145) | 0.203 | 0.217 | 0.044 (-0.048, 0.137) | **0.303** | **0.308** | 0.018 (-0.159, 0.194) |
| PFAS | PFHxS | 0.088 | 0.323 | 0.002 (-0.064, 0.067) | 0.175 | 0.406 | 0.012 (-0.098, 0.123) | 0.210 | 0.262 | 0 (-0.092, 0.093) |
| PFAS | PFOA | 0.088 | 0.341 | -0.012 (-0.087, 0.063) | 0.175 | 0.233 | -0.005 (-0.102, 0.093) | 0.210 | 0.427 | -0.014 (-0.147, 0.119) |
| PFAS | PFOS | 0.088 | 0.337 | 0.018 (-0.057, 0.092) | 0.175 | 0.362 | -0.002 (-0.114, 0.11) | 0.210 | 0.311 | 0.011 (-0.107, 0.129) |
| Phenols | BPA | 0.131 | 0.580 | -0.014 (-0.09, 0.062) | 0.170 | 0.476 | -0.007 (-0.111, 0.097) | 0.249 | 0.574 | -0.005 (-0.167, 0.157) |
| Phenols | TCS | 0.131 | 0.420 | 0.05 (-0.052, 0.153) | 0.170 | 0.524 | 0.049 (-0.095, 0.192) | 0.249 | 0.426 | 0.06 (-0.111, 0.231) |
| Phthalates | MBzP | 0.144 | 0.161 | 0.062 (-0.011, 0.135) | **0.276** | **0.316** | **0.137 (0.016, 0.259)** | 0.264 | 0.072 | 0.007 (-0.073, 0.087) |
| Phthalates | MCPP | 0.144 | 0.159 | -0.025 (-0.082, 0.032) | **0.276** | **0.155** | -0.018 (-0.121, 0.085) | 0.264 | 0.120 | -0.022 (-0.094, 0.05) |
| Phthalates | MEP | 0.144 | 0.038 | 0.015 (-0.03, 0.059) | **0.276** | 0.039 | 0.009 (-0.045, 0.063) | 0.264 | 0.063 | 0.008 (-0.05, 0.066) |
| Phthalates | MMP | 0.144 | 0.093 | -0.009 (-0.05, 0.031) | **0.276** | 0.031 | 0.002 (-0.041, 0.044) | 0.264 | 0.214 | -0.014 (-0.1, 0.071) |
| Phthalates | ΣDEHP | 0.144 | 0.087 | -0.017 (-0.074, 0.04) | **0.276** | **0.195** | -0.076 (-0.187, 0.036) | 0.264 | 0.072 | 0.013 (-0.062, 0.087) |
| Phthalates | ΣDiBP | 0.144 | 0.151 | -0.042 (-0.118, 0.033) | **0.276** | 0.060 | -0.033 (-0.118, 0.052) | 0.264 | 0.188 | -0.025 (-0.159, 0.109) |
| Phthalates | ΣDiDP | 0.144 | 0.077 | 0 (-0.032, 0.033) | **0.276** | 0.051 | 0.001 (-0.048, 0.05) | 0.264 | 0.085 | 0.004 (-0.038, 0.047) |
| Phthalates | ΣDiNP | 0.144 | 0.176 | 0.007 (-0.075, 0.089) | **0.276** | **0.107** | -0.049 (-0.151, 0.053) | 0.264 | 0.112 | 0.045 (-0.051, 0.14) |
| Phthalates | ΣDnBP | 0.144 | 0.059 | -0.002 (-0.05, 0.045) | **0.276** | 0.044 | -0.002 (-0.057, 0.053) | 0.264 | 0.074 | -0.003 (-0.062, 0.057) |
| Solvents | ΣNEP | **0.278** | **1.000** | 0.084 (-0.011, 0.179) | 0.232 | 1.000 | 0.076 (-0.059, 0.212) | **0.317** | **1.000** | 0.108 (-0.08, 0.295) |
| Tobacco Met. | Cotinine | 0.112 | 1.000 | -0.006 (-0.122, 0.111) | 0.236 | 1.000 | 0.067 (-0.144, 0.277) | 0.252 | 1.000 | -0.036 (-0.228, 0.157) |
